# PROMIS® Health Organization (PHO) 2020 Conference Toward Patient-Centered Care: PROMIS Implementations and Advances Abstracts: Virtual. 25-27 October 2020

**DOI:** 10.1186/s41687-020-00250-5

**Published:** 2020-11-18

## 101-O. Developing PROMIS-based research and clinical profiles for patients with heart failure

### Faraz S. Ahmad, Kathryn L. Jackson, Leilani Lacson, Susan E. Yount, Nan E. Rothrock, Michael A. Kallen, Karl Y Bilimoria, Abel N. Kho, David Cella

#### All authors: Northwestern University Feinberg School of Medicine, Chicago, Illinois

##### **Correspondence:** Faraz Ahmad (faraz.ahmad@northwestern.edu)

**Objectives**

Heart failure (HF) is a common and morbid condition. We previously reported the development of the PROMIS®-Plus-HF Profile Measure, a complete assessment of health that combines generic and HF-specific items. To facilitate patient-centered research and care, we sought to develop research and clinical profiles of the PROMIS-Plus-HF measure with overall, physical, mental, and social summary scores.

**Methods**

Candidate items (n=31) for the Research and Clinical Profiles were selected from the 86 items in the PROMIS- Plus-HF Profile Measure based on psychometric properties and to ensure coverage of the range of symptoms experienced by HF patients. In a web-based survey, HF clinicians (n=43) rated item importance and clinical actionability. Informed by these results, the study team developed a 27-item Research Profile and 10-item Clinical Profile. Overall, physical, mental, and social health summary scores on a scale of 0 to 100 were calculated. In a cross-sectional (n=600) sample, we measured the reliability: internal consistency with Cronbach’s alpha and test-retest in sample of 100 participants. Known groups validity was assessed using one-way ANOVA, modeling difference in HF score across New York Heart Association (NYHA) Class and Kansas City Cardiomyopathy Questionnaire (KCCQ) summary scores and subscales. Differences in change in Profile scores (responsiveness) by KCCQ change was evaluated using linear mixed regression.

**Results**

In the 600-person cross-sectional sample, the summary scores for the 27-item PROMIS-Plus-HF Research Profile and 10-item Clinical Profile were normally distributed. Internal consistency for domain scores were excellent (all α >0.8). Test-retest intraclass correlation coefficients for domain and overall scores were ≥0.90. Both profiles demonstrated known groups validity for overall score and physical sub-score based on NYHA Class, and for all scores based on KCCQ groups (p<0.05). In the 75-person longitudinal sample, the Research Profile demonstrated evidence of responsiveness (p<0.05) for the overall and domain scores. For the Clinical Profile, the point estimates for the overall and social and mental health scores reflected responsiveness and the change in physical score reached statistical significance (p<0.05).

**Conclusion**

The PROMIS-Plus-HF Research and Clinical Profiles demonstrated overall good psychometric characteristics. The PROMIS-Plus-HF Research and Clinical Profiles may be used to facilitate patient-centered research and clinical care.

## 102-P. Withdrawn

## 103-P. Is there a difference in outcomes between patients treated with different implants for hammertoe correction?

### Amanda Holleran, Adolf S. Flemister, Benedict DiGiovanni, Irvin Oh, John Ketz, Gabriel Ramirez, Caroline Thirukumaran, Judith F. Baumhauer

#### All authors are from University of Rochester Medical Center

##### **Correspondence:** Judith F. Baumhauer (Judy_Baumhauer@urmc.rochester.edu)

**Objective**

Hammertoe surgery is one of the most commonly performed musculoskeletal surgeries. Thirty years ago, a simple 10 cents wire was used to stabilize the repair. In the past 10-15 years a multitude of implants have been suggested to replace the simple wire technique due to recurrence rates of the deformity. With new implants comes significant cost increases. This study examines the physical function, pain, recurrent and other complications of patients treated with 4 different surgical implants for hammertoe correction.

**Methods**

A retrospective review of prospectively collected patient reported outcome measurement information system (PROMIS) physical function PF and pain interference PI data was performed in 248 patients who had a hammertoe correction January 2015-December 2019. Categorical (yes/no) for recurrence and complications was obtained by chart review. Mann-Whitney U, Chi-square test and mixed linear regression models for were used to compare groups for demographics and assess PF and PI differences at final follow up time point for each implant group (k-wire, nextra implant, retrograde fusion screw, Trim it pin) correcting for confounding demographic variables.

**Results**

Baseline demographics demonstrated implants were used in slightly older aged patients (2 years average). Other confounding variables included BMI (larger had lower PF), Smoking history (past smokers had lower PF and higher PI), insurance (governmental products had lower PF and higher PI). Implants had a higher recurrence rate (OR 1.9) however no increase in other complications. At final follow up when controlling for confounding variables, PF was better with nextra and trim-it pins than k- wire. There was no difference in PI between K-wire and implant groups.

**Conclusions**

There is variation in the surgical implants used for the commonly performed hammertoe procedure. The choice of implant should be based on patient reported outcomes (function and pain improvement) as well as the risk of recurrence or complications. In this case, the cost differential between the k-wire and the few implants reviewed is nearly 1,000 dollars. Objective assessments of outcomes will aid in determining value, eliminate variation and improve the alignment of provider and health care cost allocation.

## 104-P. Is there a difference in outcomes between double or triple arthrodesis for foot deformity?

### Amanda Holleran^1^, Judith F. Baumhauer^1^, Jeff Houck ^2^, Daniel Homeier^1^, Adolf S. Flemister^1^, Benedict DiGiovanni^1^, Irvin Oh^1^, John Ketz ^1^

#### ^1^University of Rochester Medical Center; ^2^George Fox University, Newberg, OR

##### **Correspondence:** Judith F. Baumhauer (Judy_Baumhauer@urmc.rochester.edu)

**Objective**

Triple arthrodesis (fusion of the talonavicular, subtalar and calcaneocuboid joints) has historically been considered the standard of treatment for arthritis of the hindfoot. The complications of this surgery include non-union, malunion, nerve injury, infection, and wound healing problems. Double arthrodesis (fusion of the talonavicular and subtalar joints) is capable of producing a similar reduction in motion and correction of foot deformity, however, may cause less patient morbidity due to one less joint being incorporated into the fusion procedure and less cost due to shorter operative time and fewer hardware needs. The purpose of this study is to evaluate the patient reported outcomes (PROMIS physical function PF and pain interference PI) and complication rates for surgically corrected foot deformity using a triple arthrodesis compared to using a double arthrodesis.

**Methods**

A retrospective review of prospectively collected patient reported outcome measurement information system (PROMIS) data was performed in 57 patients who had either undergone a double or triple arthrodesis from January 2015-December 2019. PF and PI scores were collected. Linear mixed models were used to assess differences over time and between groups (Double versus Triple) pre- operation, 3 months, 6 months, 9 months and 12 months post-surgery. Medical records were reviewed for complications (yes/no).

**Results**

There were no statistical differences between groups in terms of age (p=0.65), BMI (p=0.32), pre-operative diagnosis (p=0.79), ASA rating (p=0.4), or complications (p=0.49) occurred. Coefficient of variation at each time point per group varied from 11.9% to 21.8%. Both groups were significantly improved in physical function (p<0.01) and pain interference (p<0.01) without a significant difference between groups at 9 or 12 months.

**Conclusion**

Double arthrodesis can allow for similar correction of foot deformities without the increased risk of wound complication and nonunion. Both groups demonstrated a significant improvement in their PROMIS PF and PI at 1 year demonstrating either a double or triple arthrodesis is a feasible operation however a double arthrodesis may potentially save time and health care costs.

## 105-O. Methodology for selecting and evaluating items from PROMIS® Item Banks to develop novel short-form questionnaires

### Steven I. Blum^1^, Larissa Stassek^2,^ Donald M. Bushnell^2^, Sejin Lee^3^, James W. Shaw^1^, Mona L. Martin^2^

#### ^1^Bristol Myers Squibb, Lawrenceville, NJ USA; ^2^Evidera | PPD, Bethesda, MD USA; ^3^University of North Carolina, Chapel Hill, NC USA

##### **Correspondence:** Steven I. Blum (steven.blum@bms.com)

**Objectives**

PROMIS® measures can be administered via computer adaptive testing or through use of existing short-forms and profile measures. Customized short-forms can be developed by selecting items from PROMIS item banks. We describe a systematic approach for selecting PROMIS items when creating custom short-forms and for generating evidence to support content validity within specific patient populations.

**Methods**

A modified Delphi process was used to initially evaluate items from the PROMIS and PROMIS-Cancer Physical Function items banks and to reduce the number of items to be further evaluated in qualitative interviews. The Delphi panel (n=10) included both measurement experts and patient representatives who evaluated the items using predefined criteria and voted over three rounds to keep or drop each item. Retained items were subsequently evaluated in combined concept elicitation/cognitive interviews. Interviews (n=150) were planned with patients diagnosed with one of five different cancers (i.e., lung, renal, hepatocellular, melanoma, and head and neck). Interviews were conducted at multiple sites in the United States and incorporated card sorting and rating exercises, which facilitated discussion on the relevance and importance of each item and any difficulty answering. The interviews were audio recorded, transcribed, and analyzed.

**Results**

The Delphi panel evaluated 169 PROMIS Physical Function items, voting to drop 93 and retain 76 items for further evaluation in the qualitative interviews. While recruitment is ongoing, preliminary results from the interviews have provided evidence for selecting PROMIS items most relevant to patients with each tumor type. The final deliverables will include a disease-specific short-form for each tumor type and an evidence dossier suitable for submission to regulatory authorities. This methodology can be applied to other measurement systems (e.g., EORTC, PRO-CTCAE) with item banks/libraries to select subsets of items relevant to a specific target population.

**Conclusions**

Qualitative patient interviews incorporating card sorting and rating exercises can be used to select a subset of items relevant to a specific population, while simultaneously generating additional evidence to support content validity of the novel short-form measures. A modified Delphi process helped to reduce the number of items that needed to be evaluated, thus making the interviews more manageable and efficient.

## 106-P. PROMIS^®^ Paediatric Self Report Profile-25 distinguishes subgroups of children with two common paediatric knee injuries

### Chaplin, J.E.^1,2^ Danielsson, A.^3,4^, Janarv, P-M.^4,5,6^, Askenberger, M.^4,7^

#### ^1^Dept. of Pediatrics, Institute of Clinical Sciences, Sahlgrenska Academy at Gothenburg University, Gothenburg, Sweden, ^2^Swedish Association of Local Authorities and Regions (SALAR); ^3^Dept. of Orthopaedics, Institute of Clinical Sciences, Sahlgrenska Academy at Gothenburg University, Gothenburg, Sweden; ^4^Swedish Paediatric Orthopaedic Quality register (SPOQ); ^5^Capio Artro Clinic, Stockholm, Sweden.; ^6^Dept. of Molecular Medicine and Surgery, Karolinska Institute, Stockholm, Sweden.; ^7^Dept. of Women’s and Children’s Health, Karolinska Institute, Solna, Sweden

##### **Correspondence:** John Eric Chaplin (john.chaplin@gu.se)

**Objective**

To test the sensitivity of the generic PROMIS-25 and the child version of the illness- specific measure Knee Injury and Osteoarthritis Outcome Score (KOOS-Child) to two specific knee injuries which involve different symptoms and treatment regimens.

**Methods**

The qualitative control of treatment of severe knee injuries is followed by the Swedish Paediatric Orthopaedic Qu**a**lity register (SPOQ). All patients were invited to complete paper versions of the two instruments at predefined follow-ups. Data collected in 2017–2019 are presented. Analyses were made of floor and ceiling effects. Agreement between domains was tested using bivariate correlation. Sensitivity of injury and treatment outcomes was investigated using a receiver operating characteristic (ROC) curve.

**Results**

Data from 272 paediatric patients (49% female; mean age 13-years at follow-up, range: 9–14) were gathered. Diagnoses: patellar dislocation 43%; anterior cruciate ligament injury (ACL) 22%; other diagnoses 33%; unknown 1%. The missing data rate was negligible: PROMIS 0.4%, KOOS 1.6%. Ceiling effects were found in all KOOS variables. The highest correlations between the domains of PROMIS (p) and the KOOS (k) were between ‘(p)mobility’ and ‘(k)sport’ (r=0.717), between ‘(p)pain interference’ and ‘(k)pain’ (r=0.634) and between ‘(p)anxiety’ and ‘(k)QoL’ (r=-0.509). Sensitivity to diagnosis (patellar dislocation and ACL) was more pronounced in PROMIS, where ‘(p)anxiety’ and ‘(p)mobility’ had the largest AUC (0.5525 and 0.5461, respectively) of all domains in both instruments.

**Conclusions**

The expected agreements between similar domains in the two instruments were found. Our results suggest that the PROMIS-25 was more sensitive to differences in anxiety and mobility between different injury locations than the KOOS-Child. Further analysis of the differences between these instruments will help to identify the measure of first choice in registry data collection.

## 107-P. PROMIS^®^ Paediatric Self Report and Proxy Profile-25 compared to a quality-of-life instrument

### Chaplin, J.E.^1,2^, Peterson, C.^3^, Danielsson, A^4,5^

#### ^1^Dept. of Paediatrics, Institute of Clinical Sciences, Sahlgrenska Academy at Gothenburg University, Gothenburg, Sweden; ^2^Swedish Association of Local Authorities and Regions (SALAR); ^3^School of Health and Welfare, Jönköping University, Jönköping, Sweden; ^4^Dept. of Orthopaedics, Institute of Clinical Sciences, Sahlgrenska Academy at Gothenburg University, Gothenburg, Sweden; ^5^Swedish Paediatric Orthopaedic Quality Register (SPOQ)

##### **Correspondence:** John Eric Chaplin (john.chaplin@gu.se)

**Objective**

To compare the generic paediatric PROMIS profile-25 (p) with the multi-dimensional quality-of-life instrument DISABKIDS-31 (d).

**Methods**

Paper versions were administered to children aged 8-17 years and their parents, and to parents only for younger children, at the orthopaedic outpatient clinic of a university hospital. PROMIS-25 is a six-dimensional PROM profile; DISABKIDS-31 is a five-dimensional quality-of- life measure. Intraclass correlation coefficient assessed agreement between child and proxy. Multiple linear regression was calculated to predict DISABKIDS total QoL from the PROMIS profile for self-report and proxy-report. To visualize the relationships between the variables, multidimensional scaling with PROXSCAL was used.

**Results**

Data on 35 children (4-17 yrs, 60% girls) were collected, which included 17 child/proxy diads (9-17 yrs), 1 child (10 yrs) and 17 parent-reports without child self-report (4-15 yrs). The most frequent reason for visiting the clinic (77%) was leg or muscle injuries; other diagnoses included injury or deformity to the feet or back. Missing data was negligible: PROMIS 3%, DISABKIDS 4%. There was good to excellent agreement between child and parent DISABKIDS (r=0.673-0.903); poor to excellent for PROMIS (r=0.272-0.975), with (p)peer relationships (r=0.272) having the lowest agreement. Predicting DISABKIDS QoL scores, a significant regression equation was found for PROMIS self-report (F(7,9)=4,931, p=0.015) with an adjusted R^2^ of 0.632, and for proxy-report (F(7,24)=14,608, p<0.001), adjusted R^2^=0.54, with (p)physical function, (p)fatigue and (p)pain intensity being reliable predictors (p<0.001, p=0.040; p=0.020, respectively). Multidimensional scaling revealed a good separation between variables for both instruments, with the possible exception of PROMIS anxiety and depression.

**Conclusions**

The two questionnaires demonstrated mixed inter-rater reliability, with PROMIS peer relations having the lowest ICC, indicating a possible greater sensitivity to differences in child/proxy reporting in PROMIS. A large part of the variation in DISABKIDS total-QoL can be explained by the PROMIS profile scores, indicating overlap between the instruments. The most reliable predictor of total-QoL was the physical functioning/mobility variable in PROMIS. Multidimensional scaling suggests that PROMIS-25 has a better separation between domains than DISABKIDS, with the possible exception of (p)anxiety and (p)depression. Further analysis of the differences between these instruments would benefit from a larger and more diverse population.

## 108-P. Patient-centered approach to response-level missing data

### Chapman, R.

#### Department of Medical Social Sciences, Feinberg School of Medicine, Northwestern University, Chicago, USA

##### **Correspondence:** Robert Chapman (Robert.Chapman@northwestern.edu)

**Objective**

To use mixture modelling as a patient-centered method to explain item-level missing data in PROMIS measures. These methods are used to evaluate the presence of item missingness across patient demographics, conditions and treatment, and patient-reported symptom severity and impact. These results inform novel Missing Not at Random models of missingness for PROMIS measures and help guide the clinician’s selection of PROMIS measures to minimize patient non-response.

**Methods**

Data used in analyses were obtained from HealthMeasures Dataverse, including data collected as a part of the development of the PROMIS Neuropathic and Nociceptive Pain Quality measures. PROMIS measures were scored using IRT item parameters and pattern- response scoring methods. Mixture modelling analyses were conducted with patient demographics, clinical information, number of items missing and PROMIS T scores. All analyses were conducted in R statistical computing software.

**Results**

Initial results with the PROMIS Neuropathic & Nociceptive Pain Dataset show that more item- level missing data is associated with better neuropathic pain scores (as indicated by lower PROMIS Neuropathic Pain Quality T scores) and with patients who have a condition associated with nociceptive pain (rheumatoid arthritis & fibromyalgia). Conversely, less missing item-level data is associated with worse pain (indicated by higher PROMIS Neuropathic Pain Quality T scores) and with patients who have conditions associated with neuropathic pain (diabetic neuropathy & cancer chemotherapy induced peripheral neuropathy).

**Conclusion**

These results provide important guidance for researchers or regulators who may be concerned about the item-level missing data in PROMIS measures: missing data is less likely to occur with patients who have worse health-related quality of life (greater symptom severity and impacts). Missing data is also less likely when patients are answering items relevant to their condition or severity. Researchers seeking to model item-level missingness for data imputation methods should focus on missingness in patients with lower symptom severity and impact. These findings reinforce the importance of administering item content relevant to the patient, which is appropriate to either the patient’s condition or severity. Clinical users can incorporate these findings into their practice by administering condition-relevant PROMIS short forms or by administering PROMIS Computer Adaptive Tests to minimize irrelevant items.

## 109-P. Measurement of minimal disease activity in psoriatic arthritis using PROMIS-Physical Function or the Health Assessment Questionnaire-Disability Index

### Erin Chew^1, 2^, Jamie Perin^3^, Thomas Grader-Beck^1^, Ana-Maria Orbai^1^

#### ^1^Johns Hopkins University School of Medicine Division of Rheumatology, Baltimore, MD; ^2^Johns Hopkins Hospital. Baltimore, MD; ^3^Johns Hopkins University School of Public Health, Department of International Health, Baltimore

##### **Correspondence:** Erin Chew, MD (echew6@jhmi.edu or erinychew@gmail.com)

**Background**

Minimal disease activity (MDA), is a treat-to-target strategy (T2T) objective in psoriatic arthritis (PsA). MDA criteria include physical function, traditionally assessed via the Health-Assessment Questionnaire Disability Index (HAQ-DI). It is of interest to assess the performance of more current physical function instruments such as the Patient-Reported Outcomes Measurement Information System-Physical Function Profile (PROMIS-PF).

**Objectives**

To assess the interchangeability of the HAQ-DI with the PROMIS-PF in the calculation of MDA in PsA.

**Methods**

Longitudinal PsA data were collected including HAQ-DI and PROMIS-PF in a PsA cohort. MDA definitions were built substituting the HAQ-DI criterion with the PROMIS-PF short form 4a (PROMIS-PF4a) or with the PROMIS-PF computer adaptive test (PROMIS-PF Bank). We assessed agreement/accuracy between HAQ-DI based and PROMIS-PF based MDA definitions at each visit and longitudinally through the kappa statistic/ROC curve analysis.

**Results**

One hundred participants contributed 352 observations with up to five visits. Mean (SD) age was 52 (12) years, 60% were female, and 43% were in MDA at baseline. Kappa statistic for PROMIS-PF based MDA reflected almost perfect agreement with HAQ-DI MDA: kappa=0.94 (95% CI 0.90-0.97) for MDA PROMIS-PF Bank and kappa=0.90 (95% CI 0.80-0.95) for MDA PROMIS-PF4a. Higher longitudinal agreement was seen between MDA HAQ-DI and MDA PROMIS-PF Bank versus MDA PROMIS-PF4a between consecutive visits: kappa ranged between 0.81-0.94 versus 0.72-0.84, respectively (Table 1). Area under ROC curve for predicting MDA HAQ-DI was 0.97 for MDA PROMIS-PF Bank and 0.95 for MDA PROMIS-PF4a.

**Conclusions**

Excellent agreement was seen between HAQ-DI and PROMIS-based MDA definitions statically and longitudinally. The PROMIS-PF Bank and PROMIS-PF4a are accurate replacements for the HAQ-DI in calculating MDA state in PsA.

## 110-O. Interpretation of PROMIS Fatigue CAT scores in solid organ transplant recipients

### Sumaya Dano^1^, Ali Rezaeishahreza^1^, Areej Ali^1^, Nathaniel Edwards^1,^ Setareh Aghamohammadi^1^, Nasab El-Dassouki^1^, Jasleen Gill^1^, Marta Novak^2^, Susan J. Bartlett^3†^, Istvan Mucsi^1†^

^†^Susan J. Bartlett and Istvan Mucsi are co-senior authors

#### ^1^Multi-Organ Transplant Program and Division of Nephrology, University Health Network, Toronto, Canada; ^2^Centre for Mental Health, University Health Network, Toronto, ON, Canada; ^3^Center for Health Outcomes Research, McGill University, Montreal, Quebec, Canada

##### **Correspondence:** Istvan Mucsi (istvan.mucsi@utoronto.ca)

**Objective**

Relating PROMIS T-scores to functional impacts can help clinicians and patients to meaningfully interpret T-scores. Here we assess the relationship between T-scores vs the last items and responses in solid organ transplant recipients (kidney (KTRs), kidney-pancreas (KPRs) and liver (LTRs)) using the PROMIS Fatigue Computer Adaptive Test (CAT).

**Methods**

A cross-sectional, convenience sample of adult KTRs, KPR, and LTRs completed the PROMIS Fatigue CAT on an electronic data capture system (DADOS, TECHNA Institute, UHN). The number of items answered, and the unique last items administered from the PROMIS Fatigue item bank were tabulated. Final T-scores were ordered from low to high, and last questions and responses at different T-scores are reported.

**Results**

Of the 373 participants, the mean (SD) age was 53(14), 235 (63%) were male, 199 (53%) were KTRs, 46 (12%) were KPRs and 128 (34%) were LTRs. T-scores were <50 (46%), 50-60 (35%), >60 (19%).A total of 18 unique last questions were completed in this study sample. Patients with T-scores ranging from 24-40 had last questions and responses that reflected no to very little fatigue. Unique last questions to this T-score range included questions about strenuous exercise and feeling “sluggish”. Responses to these questions suggested that patients were able to perform strenuous exercises and did not feel tired. Patients with T-scores 60 had last questions and responses reflecting moderate to severe fatigue. Unique last questions administered to patients with T-scores 60 included questions about fatigue interfering with physical functioning, and for patients with T-scores >70, the ability to eat and carry a conversation. Responses to questions in this T-score range suggested that fatigue limited the ability to perform even basic daily activities of living.

**Conclusion**

We reported a relationship between PROMIS Fatigue CAT T-scores, and the last question and response administered. This relationship can help improve the interpretation of PROMIS Fatigue T-scores and help clinicians and patients understand how PROMIS Fatigue T-scores relate to limitations in daily life.

## 111-P. Reducing questionnaire burden when screening for depressive symptoms in patients with end-stage kidney disease

### Sumaya Dano^1^, Evan Tang^1^, Faisal Jamil^1^, Dean Christidis^1^, Madeline Li^3^, Doris Howell^4^, John Devin Peipert^5^, Susan J. Bartlett^6^, Istvan Mucsi^1^

#### ^1^Multi-Organ Transplant Program and Division of Nephrology, University Health Network, Toronto, Canada; ^2^Centre for Mental Health, University Health Network, Toronto, ON, Canada; ^3^Department of Supportive Care, Princess Margaret Hospital, Toronto, Ontario, Canada ^4^Princess Margaret Cancer Center, Faculty of Nursing, University of Toronto, Toronto, Ontario, Canada; ^5^Department of Medical Social Sciences, Northwestern University Feinberg School of Medicine, Chicago, Illinois; ^6^Center for Health Outcomes Research, McGill University, Montreal, Quebec, Canada

##### **Correspondence:** Sumaya Dano (sumaya.dano@mail.utoronto.ca)

**Objective**

Routine screening for depressive symptoms can be time-consuming and burdensome for patients. However, patients without depressive symptoms can be quickly screened out using ultra-brief screening tools and avoid the need of completing more precise, but longer, questionnaires. In this study we compare the questionnaire burden of completing the Patient Health Questionnaire (PHQ9) or PROMIS Depression Computer Adaptive Test (D-CAT) vs using various two-step screening combinations for depressive symptoms in patients with end-stage kidney disease (ESKD).

**Methods**

A cross-sectional, convenience sample of adult kidney transplant recipients and patients on maintenance dialysis completed the Edmonton Symptom Assessment Survey-revised (ESASr), PROMIS D-CAT and PHQ9. PHQ9 score ≥10 was used as reference to identify moderate/severe depressive symptoms. ESASr depression (ESASr-D) and PHQ2 score of ≥1 and ≥2 were evaluated for the pre-screening step. In the second step, D-CAT T-score ≥55 was used to identify patients with potentially significant depressive symptoms. The total number of questions completed were calculated for the different scenarios.

**Results**

Mean(SD) age of the 164 participants was 52(17), 68% were male, 62% Caucasian. Based on PHQ9, 16% (n=26) had depression. In the single step screening scenarios, the sample would complete a total of 1476 PHQ9 or 1020 D-CAT items, respectively (9 or 6 items per participant on average, respectively). All the different 2-step screening combinations would reduce the total number of items completed by the total sample by at least half. A 2-step method combining PHQ2 ≥2 and D-CAT (Sensitivity:65% Specificity:94%), required a total of 510 items (both PHQ2 and D-CAT together; 3.1 per participant on average). A 2-step screening combining ESASr-D ≥1and D-CAT (Sensitivity:58% Specificity:94%) required a total of 435 items (both ESASr-D and D-CAT together; 2.7 per participant on average).

**Conclusion**

Compared to administering either PHQ9 or PROMIS Depression CAT to all participants, a 2-step process including an ultra-brief pre-screening tool reduced the number of questions completed by the total sample substantially.

## 112-P. Psychometrics of three Swedish pediatric item banks from the Patient-Reported Outcomes Measurement Information System (PROMIS)^®^

### Frida Carlberg Rindestig^1^, Marie Wiberg^2^, Eva Henje Blom^1^, Inga Dennhag^1^

#### ^1^Child and Adolescent Psychiatry, Department of Clinical Science, Umeå University, Sweden; ^2^Department of Statistics, USBE, Umeå University

##### **Correspondence:** Inga Dennhag (inga.dennhag@umu.se)

**Objective**

The Patient-Reported Outcomes Measurement Information System (PROMIS®) aims to provide self-reported item banks for several dimensions of physical, mental and social health. Here we investigate the psychometric properties of the Swedish pediatric versions of the item banks for pain interference, fatigue and physical activity.

**Methods**

12-19 years old participants (n = 681) were recruited in public school settings, at a child- and psychiatric outpatient clinic, and a youth health outpatient clinic confirmatory factor analyses (CFA) were performed to evaluate scale dimensionality and local dependence. Item Response Theory (IRT) analyses were then used to finalize item banks and assure that each item is valid and weighted as a standalone assessment.

**Results**

CFA results confirmed that pain interference, fatigue and physical activity are separate constructs. Items with low item fit and items with Differential Item Functioning (DIF) were removed resulting in 14 items of pain interference and 15 items of fatigue items, and 6 items of physical activity.

**Conclusions**

Swedish Item banks were developed to assess pain interference, fatigue and physical activity in 12-19 year olds by using item response theory. These instrument offers precise, efficient and flexible assessment and allow researchers to select only the most useful items to study.

## 113-P. Parental caregiver burden and recovery of adolescent Anorexia nervosa after multi-family therapy

### Inga Dennhag, Eva Henje Blom, Karin Nilsson

#### All authors: Child and Adolescent Psychiatry, Department of Clinical Science, Umeå University, Sweden

##### **Correspondence:** Inga Dennhag (inga.dennhag@umu.se)

**Objective**

Parental involvement in the treatment of anorexia nervosa has shown to be extremely important, especially for adolescents. This study investigated whether parental caregiving burden changed during adjunct multi-family therapy of adolescent anorexia nervosa and eating disorders not otherwise specified (EDNOS) and whether caregiver burden at baseline and changes in caregiver burden during treatment were associated with treatment outcome.

**Methods**

Twenty-four females, 13 to 16 years old, and their parents, participated in the study. Caregiver burden was measured with the Eating Disorders Symptom Impact Scale, by mothers (n=23) and fathers (n=22). Treatment outcome was measured by adolescent body mass index, level of global functioning and self-rated eating disorder symptoms by the Eating Disorders Examination Questionnaire 4.0.

**Results**

All patient outcomes improved and overall caregiver burden decreased significantly during treatment. When broken down in aspects of caregiver burden the decrease in parental perceived isolation, was found to be associated with improvement of BMI and Children’s Global Assessment Scale. When analyzing fathers and mothers separately, we found that maternal feelings of guilt and paternal perceived burden of dysregulated behaviors at base-line were correlated to treatment outcome.

**Conclusions**

Multi-Family Therapy shows preliminary effectiveness as an adjunct treatment for anorexia nervosa and eating disorders not otherwise specified. Fathers might be more important than seen before in treatment, especially in the participation of Multi-Family Therapy. Caregiver burden can be a potential mediator of treatment results in the future.

## 114-P. Measuring function in a multidisciplinary Osteogenesis Imperfecta clinic

### Maureen Donohoe, Cristina McGreal, Jeanne M. Franzone, Richard W. Kruse, Michael B. Bober, Kenneth Rogers, Robert Wellmon

#### All authors: Nemours/ Alfred I. duPont Hospital for Children, Wilmington, DE

##### **Correspondence:** Maureen Donohoe (Reenee.Donohoe@nemours.org)

**Objective**

Our objective is to report on early results of data collected during multidisciplinary clinic visits using PROMIS, functional mobility scores (FMS), and BMI, identifying relationships between type of Osteogenesis Imperfecta (OI) and function.

**Methods**

This is a single center retrospective review of OI patients attending a clinic visit including Genetics, Orthopaedics, and Physical Therapy between January, 2016– October, 2019. Demographic, clinical, operative data, PROMIS dimensions including physical mobility, upper extremity function, pain interference, fatigue, and peer relationships (pediatric) or social participation (adult) and FMS were collected. Individuals’ presentations were sorted by mild, moderate, or severe and by BMI into categories of ideal, overweight, and obese.

**Results**

49 met criteria and were grouped based on OI severity. OI severity was associated with higher BMI and lower levels of function on PROMIS Physical Mobility and Upper Extremity Function dimensions. BMI was negatively associated with PROMIS Physical Mobility score. Individuals with OI who scored higher on PROMIS Physical Mobility and Upper Extremity Function had lower levels of Pain and Fatigue based on reported scores. Statistical significance between group differences for BMI, and PROMIS scores for Physical Mobility and Upper Extremity Function. Participants with mild or moderate OI severity had significantly lower BMI than those with severe OI. PROMIS Physical Mobility: participants with mild and moderate OI had significantly higher scores than those with severe OI; individuals with mild OI also scored significantly higher than those with moderate OI severity. PROMIS Upper Extremity Function: participants with mild OI had significantly higher scores than those with moderate or severe OI.

**Conclusions**

Patient reported outcome (PRO) measures are helpful in understanding individuals’ functional levels and identifying needs. Mild OI presentation tend to have lower BMI and greater activity as noted on PROMIS. Fatigue and Pain Interference on PROMIS did not have a significant relationship based on severity of OI or BMI. Severe presentation of OI tend to have higher BMI and less physical activity and upper extremity function on PROMIS. Past year fracture history, surgical intervention, and bisphosphonate use had no statistically significant impact on PRO across this population.

## 115-O. Cross-walking PROMIS-29 to the Roland-Morris Disability Questionnaire and Oswestry Disability Index for chronic back pain

### Maria Orlando Edelen^1^, Anthony Rodriguez^1^, Patricia M. Herman^1^, Ron D. Hays^1,,2^

#### ^1^RAND, ^2^UCLA

##### **Correspondence:** Maria Orlando Edelen (orlando@rand.org)

**Objective**

There is extensive literature on the effectiveness of pharmaceutical and nonpharmacologic interventions for chronic low back pain (CLBP) based on different samples and outcome measures. The NIH Research Task Force (RTF) on CLBP noted that these differences make it difficult to compare studies of similar or competing interventions. These differences limit the usefulness of the results in answering questions such as ‘Which therapies work best? And for whom?’ This study reports empirical links of the PROMIS-29 with the Roland-Morris Disability Questionnaire (RMDQ) and the Oswestry Disability Index (ODI) to enable comparisons across more studies.

**Methods**

Secondary analyses of three datasets: 1) RAND Center of Excellence for the Appropriateness of Care (CERC) data (n=1677) were collected on chiropractic patients being treated for CLBP and CNP; 2) Assessment of Chiropractic Treatment for Low Back Pain (ACT) data (n=750) were collected on active military personnel participating in chiropractic clinical trials for LBP; and 3) Amazon Mechanical Turk (MTurk) data were obtained from a general population sample (n=5755) that included a subgroup that reported CLBP (n=1444). The PROMIS-29 was administered in all three datasets, the RMDQ in the ACT, and the ODI in the CERC and MTurk datasets. We develop ordinary least squares regression equations to predict the RMDQ and the ODI from PROMIS-29 scales.

**Results**

*R*^*2*^ values ranged from 54 to 61% with normalized mean absolute error (NMAE) ranging from 0.51 to 0.53 standard deviations in regression models predicting the RMDQ from the PROMIS-29. Physical function, pain interference, and sleep disturbance were consistently retained. *R*^*2*^ values ranged from 65 to 67% in CERC data and 63% in MTurk data with NMAE ranging from 0.43 to 0.47 in CERC and 0.46 in MTurk data for predicting the ODI. Physical function, social function, sleep disturbances, and average pain intensity were consistently retained.

**Conclusions**

The RMDQ and ODI “legacy” scores can be predicted from the PROMIS-29 with sufficient accuracy for group-level comparisons. These crosswalks enable comparisons of studies that use legacy measures with those that administer the PROMIS-29. In addition, these results can be used for the harmonization required for individual patient data meta-analyses.

## 116-O. Dutch reference values for the PROMIS Scale v1.2 – Global Health

### Ellen BM Elsman^1^, Leo D Roorda^2^, Martine HP Crins^2^, Maarten Boers^1^, Caroline B Terwee^1^

#### ^1^ Amsterdam UMC, Vrije Universiteit Amsterdam, Department of Epidemiology and Biostatistics, Amsterdam Public Health research institute, Amsterdam, the Netherlands ^2^ Amsterdam Rehabilitation Research Center | Reade, Amsterdam, the Netherlands

##### **Correspondence:** Ellen Elsman (e.elsman@amsterdamumc.nl)

**Objective**

In order to add context to the health impact of diseases and conditions, it is important to interpret and compare patient-reported outcomes across studies and populations. This study aims to estimate and evaluate Dutch reference values for the Patient- Reported Outcomes Measurement Information System Global Health (PROMIS-GH) scale.

**Methods**

The PROMIS-GH v1.2 was administered through a web-based survey to 4370 Dutch persons, representative for the Dutch general population in 2016. T-scores for the mental health (GMH) and physical health (GPH) subscales, and their shorter two-item subscales, were calculated for the entire population, age groups and gender. T-scores for GMH and GPH were compared to the US reference population, which has a mean T-score of 50 and a standard deviation of 10, and to age-range and gender subpopulation reference scores. US reference population T-scores are representative for the 2000 US general population.

**Results**

The Dutch population had a GMH T-score of 44.7 and a GPH T-score of 45.2, both substantially lower, and thus worse, than the US reference population T-score of 50. Lower T-scores for the Dutch general population were found for both age-range and gender subpopulations compared to US subpopulation reference values. T-scores of the Dutch general population showed a similar pattern compared to US reference values: T-scores worsened with increasing age, but improved again for the oldest age groups; males scored better than females.

**Conclusions**

This study reports reference values for the PROMIS-GH scale for the Dutch general population, including age-range and gender subpopulations. PROMIS can improve the assessment of physical and mental health, but appropriate population reference values are essential for their interpretation. This study provides these values for the Netherlands; they are notably worse from the US reference values of 2000; perhaps the US data is outdated and no longer representative of the current US health status. Nevertheless, this study fuels the discussion on whether or not we should anchor the mean and standard deviation of PROMIS scales on the US population.

## 117-P. PROMIS in English speaking countries – A systematic review of the evidence for measurement invariance of PROMIS tools

### Alex Matthews, Jonathan P Evans, Jose Valderas

#### All authors: Health Services and Policy Research Group, University of Exeter Medical School, Exeter, UK

##### **Correspondence:** Jonathan P Evans (j.p.evans2@exeter.ac.uk)

**Objective**

Measurement invariance across different populations defined in terms of language and culture must be quantified and confirmed to ensure that Patient Reported Outcome Measures (PROMs) maintain their metric properties. The Patient Reported Outcomes Measurement Information System (PROMIS) was designed and tested on a US reference population. Assumptions of validity and cross-cultural equivalence in other English-speaking countries is based on a universal translation approach, but remains untested and should be confirmed alongside evaluation of other psychometric properties such as reliability and responsiveness. We aimed to investigate the use of PROMIS instruments in non-USA English speaking countries, and the evidence of measurement invariance within these populations.

**Methods**

We performed a systematic search of MEDLINE and Embase for contemporary literature from 2017 onwards. Articles were included if they provided evidence of use or assessment of metric properties of PROMIS instruments in UK, Australian or New Zealand populations. Secondary searches of published abstracts from conference proceedings and trial registries were also undertaken.

**Results**

Twenty-two articles met our inclusion criteria and 12 (55%) used a PROMIS instrument as an outcome measure without any evaluation of their metric properties in the target populations. The remaining 10 articles analysed the metric properties of PROMIS tools. Six Australian psychometric analyses focused on mental health metrics for the Depression, Anxiety and Emotional Distress item banks. Three studies provided evidence to support validity, responsiveness to change was confirmed in two and measurement invariance was assessed in one. Only four studies including UK populations studied either the validity, responsiveness or invariance. Sixty-nine registered clinical trials were identified. The majority planned to use PROMIS tools to assess outcomes. There was no evidence of cross-cultural adaptation or testing for cross-cultural equivalence of PROMIS item banks.

**Conclusion**

Evidence on the measurement properties of PROMIS instruments in populations from English speaking countries outside of the US and Canada is sparse. Lack of confirmation of measurement invariance places the interpretation of PROMIS instruments at risk. There is a pressing need for the evaluation of cross-cultural validation amongst English speaking populations to ensure appropriate interpretation and acceptance of the PROMIS instruments.

## 118-O. Monotonic polynomials to model flexible item response curves for PROMIS Physical Function

### Carl F. Falk^1^, Felix Fischer^2^

#### ^1^Department of Psychology, McGill University, Montréal, Québec, Canada; ^2^Department of Psychosomatic Medicine, Center for Internal Medicine and Dermatology, Charité – Universitätsmedizin Berlin, corporate member of Freie Universität Berlin, Humboldt-Universität zu Berlin, and Berlin Institute of Health, Berlin, Germany

##### **Correspondence:** Felix Fischer (felix.fischer@charite.de)

**Objective**

The PROMIS Instrument Development and Validation Scientific Standards suggest to investigate each items’ measurement properties by inspecting initial probability functions from non- parametric IRT models. Typically, items are excluded when their response function is misfitting a parametric model. Monotonic polynomials allow to parametrically model aberrant response curves and therefore to retain such items in the measurement model. We investigated suitability of this approach in the PROMIS Physical Function item bank.

**Methods**

Using PROMIS Wave 1 data (N = 15,725) for Physical Function, we fitted a monotonic polynomial model as well as the standard graded response model. We compared both models in terms of overall model fit, latent trait estimates, and item as well as test information. We investigated item-level differences between both models using common measures of differential item functioning and simulated the impact of model differences on scoring of 5 and 10 item tests.

**Results**

The monotonic polynomial showed better fit to the data indicated by a significant likelihood ratio test and a lower AIC (but higher BIC) compared to the graded response model. The difference of theta estimates between both models was less than 0.12 in 95% of the cases, but the monotonic polynomial model had higher information in the lower ranges of the construct. The high concordance between both models could be due to the fact that items with aberrant response curves have not been included in the PROMIS Physical Function itembank.

**Conclusions**

Monotonic polynomials as flexible intermediates between parametric and non- parametric models appear to be a useful addition to PROMIS developers’ toolbox.

## 119-P. Development and pilot testing a self-reported pediatric PROMIS app for young children aged 5-7 years

### Wenjun Gao, Changrong Yuan, Yuchen Zou, Huan Lin

#### all authors: School of Nursing, Navy Medical University, Shanghai, China.

##### **Correspondence:** Wenjun Gao (zerowenjun@163.com)

**Objective**

The aims of this study are threefold. Firstly, using the state of science PROMIS (Patient-Reported Outcomes Measurement Information System) methods to develop a smartphone application to monitor the emotional distress for young children aged 5-7 years old; Secondly, to test the usability of this application; and thirdly, to determine the level of agreement between reports by parents and young children's self-report.

**Methods**

A multidisciplinary research team, made up of senior pediatric nurses and doctors, software engineers' team, and pediatric health researchers worked together to develop this application. Three phases of stakeholders and user studies were conducted. Phase 1 focused on prototype development; Phase 2 involved cognitive interview and usability testing; Phases 3 focused on the pilot testing of this application.

**Results**

We included the original parent proxy reporting version of Patient Reported Outcome Measurement Information System-emotional distress in the application, as well as self- reporting animated version for young children. After many rounds of modification, all participants felt that this application was easy to use and the animated items were easy to understand for young children aged 5-7 years. Correlations between parents-children reports are significant and moderate, parents underestimated child depression, and overestimated child anger and anxiety compared to child self-report.

**Conclusions**

This smartphone application and its Web-based administration portal demonstrate good usability and are well accepted by young children aged 5-7 years, which can be used to promote young children's participation when reporting or assessing symptoms of young pediatric patients. Parent reports cannot be substituted for child reports and evaluations of pediatric patients' perspectives regarding treatment outcomes should be included in pediatric clinic. This animated application can be used as a smart measurement to investigate the symptoms for young children aged 5-7 years, so as to amplify young children's voice in clinical care.

## 120-P. Difficulties in conducting online surveys among children and adolescents using translated Short Form of the PROMIS Ped SF v2.0 – Depressive symptoms 8b and modified Korovessis questionnaire

### Emilia Wołyniec ^1^, Bożena Glinkowska.^2^, Wojciech Glinkowski ^3,4,5^

#### ^1^Department of Rehabilitation, Medical University of Warsaw, Poland; ^2^Department of Sports and Physical Education, Medical University of Warsaw, Poland; ^3^Polish Telemedicine and eHealth Society, Warsaw, Poland; ^4^Center of Excellence "TeleOrto" for Telediagnostics and Treatment of Injuries and Disorders of the Locomotor System, Medical University of Warsaw, Warszawa, Poland; ^5^ Polish PROMIS National Center, Warsaw, Poland

##### **Correspondence:** Wojciech Glinkowski (w.glinkowski@gmail.com)

**Introduction**

Surveys are one of the basic and commonly used measuring tools to describe the phenomenon of interest to us. A quick and straightforward to implement online form facilitates and shortens the time of the entire process. Tests and research instruments must meet the criterion of reliability. The study aimed to determine the difficulties that should be considered in conducted surveys among children and adolescents based on questionnaires about body posture, physical activity, back pain, and symptoms of depression.

**Aim of the study**

The research was carried out at randomly selected schools in Warsaw and Tczew. The study involved 85 teenagers attending elementary school classes.

**Material and methods**

The study was conducted in 2 groups (32 participants - average age 12.3 years and 53 participants average age 11.8 years). The study was conducted using the internet "mini-questionnaire" http://mini-ankieta.azurewebsites.net/ with the consent of the Bioethics Committee. In both groups, the study was conducted twice, with the second one after a one-week break, as recommended for the reliability studies. The questionnaire consisted of 53 items. Questions include data on age, weight, and height as well as on carrying a backpack/school bag, school and sports activity, and the presence of posture defects (Korovessis, Glinkowska). Besides, the PROMIS Ped SF v2.0 – Depressive Symptoms 8b (eight items) was used to assess the participants' mood for back and neck pain. The groups differed in information resources during the procedure of signing informed consent (standard vs. enriched with additional instructions and introduction to the problem of back and neck pain and problems with posture). Retest test reliability testing was performed, and Cronbach's alpha values were calculated using Medcalc version 19.1 software.

**Results**

In both groups, reliability in the questions asked sex, body build, and basic data from everyday life (e.g., backpack weight, number of hours spent on various school activities and outside school) showed good Cronbach's alpha results (> 0.7). In the group in the standard procedure, Cronbach's alpha values were insufficient (from 0.1 to 0.56), especially questions about sadness, weakness, fatigue, and exhaustion. Student information about themselves was highly consistent (Height - alpha 0.97; Weight 0.86). In the second group, data from 53 students about themselves were good - Cronbach's alpha> 0.7.

**Discussion**

The too-short range of information provided before testing among children and adolescents may result in low compliance of the responses in the test-tester, which could affect the reliability of the research instrument. Analysis of potential causes suggests that among the reasons there may have been motivational problems for the scrupulous and faithful answering of questions by children and adolescents.

**Conclusions**

Research confirms the need to inform children accurately and young people about issues related to surveys; otherwise, there is a risk of unreliable research.

## 121-O. Feasibility using PROMIS-CAT in a sports medicine center and regenerative medicine registry in outpatient setting

### Marc Gruner, Mark Nyman, Kelsey Wolff, Jacob Sellon, Karina Gonzalez

#### All authors: Mayo clinic Rochester, MN

##### **Correspondence:** Marc Gruner (grunerm@gmail.com)

**Background**

PROMIS-CAT is a patient reported outcome (PRO) tool used to assess the health status of patients. Prior to working on this pilot, many challenges existed for PRO collection. For example, patients coming to the sports medicine center, there were no universal PRO. Additionally, a regenerative medicine registry existed at the sports medicine center for ambulatory procedures via third party software (TPS). The regenerative registry had a low percentage follow-up outcome response and select providers were able to utilize the registry. This pilot aimed to evaluate the completion rate of PROMIS questionnaires among patients presenting for outpatient evaluation to the Sports Medicine. A second aim was to compare the completion rate of follow-up data of PROMIS/Epic data implementation to legacy measures/TPS.

**Methods**

PROMIS-CAT was implemented via the EHR using patient online services (POS) portal. The first aim consisted of collecting PROMIS-CAT Pain Interference (PI) and Physical function (PF) as the instruments for measuring outcomes on all patients coming to the sports medicine center. The second aim consisted of autonomously identifying ICD-10 codes prior to a regenerative procedure as lower body vs. upper body procedure. For a lower body procedure, PROMIS-CAT PI/PF was used. For an upper body procedure, PROMIS-CAT PI/UPF was used. Baseline and Follow up outcome measures were sent after a procedure at 6 weeks, 3 months, 6 months, 1 year, and 2 years.

**Results**

A review was performed monthly to assess evaluation of the first aim. 728/1028 patients seen in the Sports Medicine center completed PROMIS measures during one month for a completion percentage of 76%. A second aim was comparing regenerative registry data after a procedure via PROMIS/EHR to legacy measures/ TPS. At baseline, 95% of patients completed PROMIS measures to 83% for TPS. Six week data had 61% completion percentage of PROMIS compared to 52% of TPS. At 3 months, 53% completed PROMIS measures compared to 43% TPS. The PROMIS registry collected 57 procedures compared to 7 in the TPS during a three-month review.

**Conclusion**

EHR linked PROMIS had higher completion rates and allowed for tracking of significantly more procedures than the TPS. Using PROMIS-CAT via the EHR for registries can improve capture rate.

## 122-P. Digital application was superior to physical therapy for orthopedic knee injuries assessed by PROMIS® measures

### Marc Gruner, Jacob Sellon, Ike Hasley, Jared Hoffmann, Karina Gonzalez

#### All authors: Mayo Clinic Rochester, MN

##### **Correspondence**: Marc Gruner (grunerm@gmail.com)

**Background**

Knee pain is one of the most prevalent musculoskeletal disorders in the US. Physical therapy (PT) is often the initial treatment for conservative care. Efficacy of a PT exercise program delivered via a digital application (Limber Health app) compared to standard PT has not been thoroughly assessed. The use of PROMIS® measures for PT in orthopedic knee injuries is limited. The aim was that Digital Home-Exercise Therapy Application (DETA) will be superior to the standard of care (PT) after 8 weeks with respect to improvement in PROMIS® pain (PI) and function (PF) Computerized Assessment Test (CAT) measures.

**Methods**

This was a multi-center, prospective; single-blind randomized clinical trial comparing PT to DETA. A total of 60 patients prescribed PT were randomly assigned. The PT groups were assigned to therapy twice a week for 8 weeks. The DETA group was assigned to 15-25 minute videos 3 times a week for 8 weeks that were tailored based on the patient’s disability and health status. The DETA’s algorithm adjusted the intensity of DETA’s program progression based on results from a 4-week interim follow-up measuring changes in PROMIS® scores. The primary outcome was change in PROMIS® scores. Patients were reviewed at baseline and at 8 weeks.

**Results**

Thirty patients completed the 8 week intervention (17 control, 13 treatment) at the time of submission. No differences existed between the groups in age or gender (p>.05). Preliminary analysis suggests changes in PI (control: -1.8±7.8, Limber app: -6.3±6.7) and PF (control: 0.46±6.6, Limber app: 5.7±7.0). Independent *t*-tests revealed absolute changes in PROMIS Physical Function were significantly greater in the DETA group compared with control, indicating a greater improvement in function; a large effect size was noted (p<.05, Hedge’s *g* = 0.77). Changes in Physical Function and Pain Interference surpassed MCID in the Limber group, but not in the control group.

**Conclusion**

An 8-week DETA program was superior to the standard of care of PT program at the time of submission. The study supports that a DETA could have similar outcomes with respect to pain and function compared to PT. This study describes an innovative approach to risk stratify patients to appropriate exercise based off of their disability.

## 123-P. PROMIS and PROs in the Symptoms System - Visualizing health in clinical care

### Emelie Gustafson^1^, Martin Wohlin^1^, John Eric Chaplin^2^

#### ^1^Uppsala University, Uppsala, Sweden; ^2^ University of Gothenburg, Gothenburg, Sweden

##### **Correspondence**: Emelie Gustafson (emelie.gustafson@symptoms.se)

**OBJECTIVES**

This presentation will explore means to: Equalize asymmetry between needs and expectations in health care using patients’ perceptions about symptoms, functions and quality of life; Balance knowledge and preferences in point-of-care interactions, leading to better outcomes and enhanced value in health care; Impower patients to take responsibility for quality of care with scientifically based methods to contribute to safer, more efficient and equal care; Implement PROMIS and other PROM instruments in a patient-driven digital system, where the combination and visualization of PROMIS measures together with other PROMs facilitates usage, with benefits for both clinical care and patients.

**METHODS**

An evaluation protocol designed according to universal and co-design principles will be described. This will explore how to visualize results and combine PROMIS measures with other PROs, facilitate long-term implementation, support patient empowerment, self- management, and improve clinical care. A mixed-methods approach will be used to explore patient and multidisciplinary perspectives on the visualization of data, and the feasibility of implementation in clinical care and for patient self-management.

**RESULTS**

Measuring patient reported outcomes (PROs) with standardized questionnaires is a scientifically sound method to gain insight into patients’ symptoms, functions and quality of life. In certain contexts, PRO collection has been linked to increased survival, improved symptom management, and good treatment results in randomized studies. PROMIS provides a set of person-centered measures that evaluates and monitors physical, mental, and social health. With its generic approach, and possibilities for modern methods of administration, it offers great advantages over historical paper questionnaires and facilitates use at many stages both for clinical care and patients. This protocol will explore how to combine and visualize PROMIS measures together with legacy questionnaires.

Processes to visualize data for patients as well as clinicians, while upholding the quality of the data collected, will be explored. In the presentation we will illustrate the visualizations tested.

**CONCLUSIONS**

Equalizing asymmetry between needs and expectations of PROs visualization for clinicians and patients requires careful consideration of the overall purpose of the data and health management.

## 124-P. Do PROMIS measures correlate with fitness and satisfaction with social roles in participants of a university wellness clinic?

### ^1^Jeff Houck,^1^Dan Kang, ^2^Mary Imboden

#### ^1^School of Physical Therapy, George Fox University, Newberg, Oregon; ^2^School of Exercise Science, George Fox University, Newberg, Oregon

##### **Correspondence**: Jeff Houck (jhouck@georgefox.edu)

**Objective**

Studies determining the concurrent validity of patient reported outcomes and performance outcomes are useful for application to clinical care. To determine the correlation (bivariate and multivariate) between a set of biopsychosocial PROMIS measures with 1) physiologic measure (VO2 Max) of fitness and 2) Satisfaction with Social Roles in attendees of a University Wellness Clinic.

**Methods**

From January to March 2020, 44 of 58 attendees (age=23.7±9.6 y.o., VO2 max=42.6±8.3 ml/kg/ml) of a University Wellness Clinic completed PROMIS computer adaptive tests (physical function [PF], pain interference [PI], fatigue, self-efficacy [SE] of managing emotions, SE of managing social, anxiety, depression and satisfaction with social roles[SSR]) and short forms (SE of daily activities [SF8]) in addition to physiologic testing (i.e. VO2 Max). Univariate correlations and multivariate linear analysis were used to assess the convergence of age, gender, and different PROMIS measures with 1) VO2 max and 2) PROMIS SSR.

**Results**

Age (r= -0.31, p=0.02), PF (r=0.46, p<0.01) and fatigue (r=-0.40, p<0.01) showed significant univariate convergence with VO2 max. Younger age, higher physical function and lower fatigue correlated with higher VO2 max values. A multivariate model including age (p=0.05), PROMIS PF (p=0.05), fatigue (p<0.01), and PI (p=0.04) resulted in a r-value of 0.62 for predicting VO2 max. Age (r= -0.40, p<0.01), PROMIS PF (r=0.44, p<0.01), PI (r=-0.44, p<0.01) and SE daily activities (r=0.35, p=0.02) showed significant convergence with SSR. Younger age, higher physical function, lower PI and higher SE with daily activities correlated with higher SSR values. A multivariate model including PROMIS PF (p<0.01), depression (p=0.05), and SE of emotions (p=0.02) resulted in a r-value of 0.56 for predicting SSR.

**Conclusions**

Perceptions of function detected by PROMIS measures associated with physical health rather than psychosocial health show better convergence with fitness in mostly younger people attending a Wellness Clinic. In contrast, measures of physical health (PF) and mental health (depression and SE emotions) showed convergence with satisfaction with social roles. These outcomes support the use of PROMIS measures of physical health to counsel young participants seeking to improve fitness and a combination of physical and mental health measures when focusing on social roles.

## 125-P. Is unacceptable self-efficacy associated with unacceptable physical health domain function and symptoms?

### Houck, Jeff^,^Kang, Dan, Philbrook, Li-Zandre, Jacobson, Ryan

#### All authors: George Fox University, Newberg, OR, United States

##### **Correspondence:** Jeff Houck (jhouck@georgefox.edu)

**Objective**

Interpretation and application of the Patient-Reported Outcomes Measurement Information System (PROMIS) Self-Efficacy for Managing Symptoms (SEsx) for orthopedic physical therapy patients is unclear. Self-efficacy is theorized to mediate PROMIS physical domain measures such as pain interference (PI), physical function (PF) and fatigue. However, no current studies document the association between acceptable levels of physical domain measures and self-efficacy. Although there are several self-efficacy measures, managing symptoms is thought to be the most applicable to orthopedic patients. The purpose of this analysis was to evaluate the associations between unacceptable SEsx with physical health domain measures (PF, PI, and Fatigue).

**Methods**

PROMIS computer adaptive tests (PF, PI, Fatigue, SEsx) were administered at initial evaluation(n=199) for spine (44.7%), lower extremity (35.7%), upper extremity (17.6%) and other reasons (2.0 %) in physical therapy. Unacceptable T-scores were coded (0,1): PF < 40, PI> 60, Fatigue>55, SE<45. Odds ratios (OR) and 95% confidence intervals (CI) were calculated to examine the associations of unacceptable SEsx with other unacceptable PROMIS measures. A logistic regression model including age, gender, unacceptable PROMIS PF, SEsx, and Fatigue was evaluated for ability to independently predict unacceptable PROMIS PI.

**Results**

Patient (age=42.5 (19.5), 60% female). The proportion of patients with unacceptable symptoms were: PF 33.5%; PI 52.5%; Fatigue 40.7%, and SEsx 46.7%. The proportion of patients with *any* unacceptable symptoms was 69.7%. A total of 14.6% reported all symptoms at unacceptable levels. Unacceptable SEsx was significantly associated with: unacceptable PI (OR = 8.3, CI 4.4 to 15.7), unacceptable PF (OR=7.5, 95%CI 3.8 to 14.9), and unacceptable Fatigue (OR=3.5, CI 1.9 to 6.2). Logistic regression showed that unacceptable PF (OR 8.20, CI 2.23 to 30.86) and unacceptable SEsx (OR 4.5, CI 2.2 to 9.3) were independent predictors of unacceptable PI.

**Conclusion**

The strong association of SEsx with PF and PI, and prevalence of unacceptable SEsx measures suggests providers should develop methods to address SEsx in patients with physical health measures indicating unacceptable function and symptoms. This finding supports the theory that addressing patient confidence and beliefs (SEsx) may enhance care directed at physical health.

## 126-P. Estimating power for clinical trials with PROMIS endpoints using Item Response Theory

### Jinxiang Hu, Yu Wang

#### ^1^University of Kansas Medical Center

##### **Correspondence:** Jinxiang Hu (jhu2@kumc.edu)

**Background**

Patient reported outcomes (PRO) are important in patient-centered health outcomes research, epidemiological studies, quality of life (QOL) studies, and clinical trials. Patient-Reported Outcomes Measurement Information System (PROMIS) is a set of standardized, generic PRO questionnaires developed for clinical and research purpose. In clinical trials, it is crucial to estimate power to avoid waste of resources while still able to detect the treatment effect. However, for clinical trials with PRO as end points, Classical Test Theory (CTT) using observed scores (e.g. total/ average scores) are routinely used for power estimation. The purpose of this project is to provide guidance for power and sample size estimate for clinical trials with PROMIS measures as endpoints using IRT.

**Methods**

Motivated from PROMIS depression scales (4a, 6a, 8a), we conducted a simulation study in order to estimate power differences between IRT- and CTT-based scoring for a two- armed prospective randomized clinical trial (control vs active arm). We simulated data using various sample size, allocation ratio, number of items, effect sizes, and missing data. Three models were fit to each simulation: IRT with MLE, IRT with Bayesian estimator, and CTT.

**Results and conclusion**

Our results showed missing data, effect size, and sample size are important indicators of IRT power. Number of items is not significantly associated with power. For rare diseases or early stage trials, it is important to use IRT framework for accurate power estimation. IRT and CTT both provides good power with large sample size and effect size. Future work can examine the IRT power for detecting change over time and non-normal distribution of latent scores.

## 127-O. Validation of PROMIS measure of itch impact and intensity in pediatric patients with Atopic Dermatitis

### Kathryn L Jackson, Jin-Shei Lai, Amy Paller, Cynthia Nowinski, Stephanie Rangel, Divya Ramachandran, Neha Puar, Vidhi Patel, Jonathan Silverberg, David Cella

#### All authors: Northwestern University, Feinberg School of Medicine, Chicago Illinois, USA

##### **Correspondence**: Kathryn Jackson (kathryn.jackson1@northwestern.edu)

**Background**

Itch is the most common symptom of pediatric skin diseases, including atopic dermatitis (AD), and greatly affects patient quality of life (QOL). Assessments of itch exist, but lack comprehensiveness and psychometric validity. To fill this gap, we have developed the new PROMIS Itch Questionnaire (PIQ-C). The PIQ-C was developed using mixed-methods approaches and consists of 45 unidimensional items, calibrated using a graded response model based on item responses from 600+ children with itch conditions. Here, we report clinical validity of the PIQ-C using cross-sectional and longitudinal data.

**Methods**

Children aged 8-17 were recruited from Chicago-area dermatology clinics. Children completed the PIQ-C and additional clinical assessments of disease severity/QOL (Itch NRS, EASI, POEM, IGA, CDLQI) at baseline and 6-month follow-up. Severity measures were categorized as mild/moderate/severe and change in severity from baseline to 6- and 12-months were calculated and categorized as improved/ same/worse change. Convergent validity was assessed by evaluating correlations of PIQ-C and an itch-related clinical measure at baseline. Known groups validity was assessed using one-way Analysis of Variance (ANOVA), modelling difference in PIQ-C score across severity group at baseline. Responsiveness to change was assessed using mixed linear regression; differences in change in PIC-Q score from baseline to six months was evaluated for differences between clinical change group.

**Results**

181 patients aged 8-17 completed baseline PIQ-C; 59 completed the 6-month follow- up. At baseline, PIQ-C was highly correlated with CDLQI (0.73), POEM (0.64), and moderately correlated with Itch NRS (0.54). Significant increase in PIQ-C was found as severity of AD increased across all clinical measures used to define severity (p<0.05 for all). The PIQ-C was responsive to change across time; patients with improved clinical score also had a significantly improved PIQ-C, and the change in PIQ-C differed across improved/same/worse change groups in the expected direction (p<0.0001 for all).

**Conclusion**

The PIQ-C measure includes aspects of itch important to assessing overall symptoms and impact. Correlations with known measures, ability to distinguish among severity groups, and responsiveness across time suggest clinical validity. Next steps include evaluating replicability of results in patients from other clinics and validation in children with other itch conditions.

## 128-P. Investigating parameter stability in the presence of high slopes

### Aaron J Kaat^1^, Stein Arne Rimehaug^2^

#### ^1^Northwestern University, Chicago IL; ^2^University of Oslo, Norway

##### **Correspondence**: Aaron Kaat (aaron.kaat@northwestern.edu)

**Objective**

There is a growing recognition that large slopes in IRT models are not as desirable of a trait as originally believed. Larger slopes suggest greater information and thus higher reliability and a shorter computer adaptive testing experience; however, slopes may be inflated when the IRT model fails to account for locally-dependent item subsets, or when there is a preponderance of individuals at the floor or ceiling of the domain. The objective of this study was to investigate the sampling distribution of the PROMIS® Pain Interference 8-item short form (where slope inflation may be occurring) using data from PROMIS 1 Wave 1, using both a standard normal latent distribution and when estimating the latent distribution using Davidian curves.

**Methods**

We utilized general population data from PROMIS 1 Wave 1, for participants with item-level data on at least 5 of the 8 items from the Pain Interference short form. In order to investigate the effect of sample size on parameter stability, we conducted a bootstrap resampling of sample size 500, 750, and 1259 (i.e., the total eligible number of participants). The primary outcome was the slope estimates across replications. We utilized factorial analysis of variance to investigate whether the slopes were significantly different by latent density, sample size, and their interaction. Each item was analyzed separately.

**Results**

There was a main-effect for sample density in all 8 items, with higher slopes with DC-IRT models. The difference by sample size was less consistent, with only 3 items showing a difference in slopes by sample size. The interaction was nonsignificant for all items.

Conclusions: Contrary to expectations, slopes were larger when the latent density was estimated using Davidian Curves. Additionally, there was a higher frequency of nonconvergence (even with 10,000 cycles) with DC-IRT models. The lack of significance for sample size was encouraging, insofar as it suggests the parameters are robust to sampling conditions. However, while the means were similar across sample sizes, the range varied more widely with the smaller sizes (as would be expected). Future research should evaluate whether a zero-inflated model would also provide consistent slope estimates as here.

## 129-P. Comparing PROMIS^®^ Global Health-10 and EQ-5D: sensitivity to clinical cut-off scores for anxiety and depression.

### Kabakibi B.^1^, Chaplin JE.^2,3^, Wicksell R.^4^

#### ^1^Dept. of Public and Global Health, Gothenburg University, Gothenburg, Sweden; ^2^Dept. of Paediatrics, Institute of Clinical Sciences, Sahlgrenska Academy at Gothenburg University, Gothenburg, Sweden; ^3^Swedish Association of Local Authorities and Regions (SALAR); ^4^Dept. of Clinical Neuroscience, Karolinska Institutet, Stockholm, Sweden

##### **Correspondence**: John Chaplin (john.chaplin@gu.se)

**Objective**

To investigate the psychometric properties of the Swedish translation of the GH-10 questionnaire.

**Methods**

PROMIS GH-10, EQ5D, GAD7 and PHQ9 were electronically collected from consecutive attendees of an emergency clinic from Sept 2018 to May 2019. Confirmatory factor analysis evaluated the two-factor structure of the GH-10: physical (PCS) and mental (MCS). Goodness-of-fit was defined as comparative fit index above .9 and standardized root mean square residual (SRMR) above 0.08. Internal consistency and discriminant validity were assessed. Analyses were repeated, stratified by cutoffs for clinical treatment, and sensitivity analysis was conducted using receiver operating characteristic (ROC) curves.

**Results**

Of 164 patients (58% female) aged 18–88 (mean: 49 years), 58% were in full-time employment; 56% were overweight or obese. The two-factor solution indicated acceptable CFI: .935, but the SRMR was .0567, thus below goodness-of-fit levels. Pain had the lowest factor score on PCS. Internal consistency for the two sub-domains was good: Cronbach’s alpha for PCS was 0.730, for MCS 0.862 and for the whole instrument 0.906. Hypothesized relationships between GH10 subdomains and the other instruments were confirmed and in line with previous published reports. Pearson’s correlations showed strong correlations of the mental health subscale to the PHQ-9 (r=0.702) and the GAD-7 (r=0.704). Moreover, the physical subscale of the GH-10 showed a good correlation with the EQ-5D index (r=0.550) and with the EQ-5D VAS (r=0.565). The area under the curve (AUC) of the MCS and PCS was higher than for EQ-5D against the GAD-7 PHQ-9 cutoffs.

**Conclusions**

Taking into account the sample size, the Swedish version of the GH10 has good psychometric properties. The less well performing item concerning pain should be investigated further.

## 130-O. A Comparison of the measurement properties of the PROMIS-Fatigue (MS) 8b against legacy fatigue questionnaires

### Paul Kamudoni^1^, Jeffrey Johns^2^, Karon Cook^5^, Rana Salem^4^, Sam Salek ^2, 3^, Jana Raab^1^, Rod Middleton^6^, Christian Henke^1^, Dagmar Amtmann^4^

#### ^1^Global Evidence & Value Development – R&D, Merck Healthcare KgaA, Darmstadt; ^2^School of Life and Medical Sciences, University of Hertfordshire, Hatfield, UK; ^3^Institute of Medicines Development, Cardiff, UK; ^4^Department of Rehabilitation Medicine, University of Washington, Seattle, USA: ^5^Feral Scholars, Broaddus, Texas, USA; ^6^UK MS Register, Swansea Medical School, Swansea, UK

##### **Correspondence**: Paul Kamudoni (paul.kamudoni@merckgroup.com)

**Objectives**

Amidst the growing number of patient-reported outcome (PRO) measures of fatigue being used in MS clinical trials and clinics, evidence-based consensus on generalizable and the most appropriate measures across different settings would be beneficial for clinical research as well as patient care.

To compare the validity and responsiveness of the *PROMIS SF v1.0 - Fatigue (MS) 8b* with the Fatigue Severity Scale (FSS) and the Modified Fatigue Impact Scale (MFIS), across US and UK populations

**Methods**

Two observational studies were performed in MS populations, as part of a PRO measure development project, including a cross-sectional study in two tertiary MS centers in the US (n=296) (US sample) and a 96-week longitudinal study in the UK MS Register cohort (*still ongoing*) (n = 384) (UK sample). Analyses included examination of: 1) relative validity based on ability to discriminate across patient subgroups according to fatigue or functional status at baseline [i.e. ANOVA-F *PRO*_*X*_ ÷ ANOVA-F *PROMIS-F(MS)8b*]; and 2) relative responsiveness, based on baseline-to-week-52 score change (Effect size) across fatigue or functional status response groups (UK sample only).

**Results**

The mean age was 44.5±11.2 / 50.7±9.4; and 74 %/ 75.9% were female (US /UK Samples). The mean *PROMIS-F(MS)8b* T-score at baseline was 57.4±10.5 / 59.9±9.4 (US sample / UK sample). Compared with the *PROMIS-F (MS)8b*, relative validity (*anchor*: GHS fatigue global question) was 86% for MFIS symptom score, 87% for MFIS total score, and 42% for the FSS. Relative to the FSS, *PROMIS-F(MS) 8b* scores were more sensitive to worsening (ES = -0.44 vs. -0.18) as well improvement (ES = 0.5 vs. 0.2) in fatigue (>=1- point increase/decrease in GHS fatigue global question) over 52 weeks of follow-up. A similar pattern of score change was observed based on other anchors.

**Conclusion**

The *PROMIS-F(MS)8b* scores showed a higher precision when differentiating levels of fatigue than the FSS or the MFIS physical or total scores, and higher responsiveness to fatigue changes than the FSS. These differences have practical implications on the application of these questionnaires in both clinical practice and research settings e.g. in sample size estimation in clinical trials.

## 131-P. Validation of the PROMIS^®^ Pediatric Item Banks Anxiety and Depressive Symptoms in a general Dutch population

### Leonie H. Klaufus^1,2^, Michiel A.J. Luijten^3,4^, Eva Verlinden^1^, Marcel F. van der Wal^1^, Caroline B. Terwee^4^, Pim Cuijpers^5^, Mai J.M. Chinapaw^2^, Lotte Haverman^3^

#### ^1^Public Health Service Amsterdam, Department of Epidemiology, Health Promotion, and Health Care Innovation, Amsterdam, Netherlands; ^2^Amsterdam UMC, Vrije Universiteit Amsterdam, Department of Public and Occupational Health, Amsterdam Public Health research institute, Amsterdam, Netherlands; ^3^Amsterdam UMC, Emma Children’s Hospital, Psychosocial Department, Amsterdam, Netherlands; ^4^Amsterdam UMC, Vrije Universiteit Amsterdam, Department of Epidemiology and Biostatistics, Amsterdam Public Health research institute, Amsterdam, Netherlands; ^5^Vrije Universiteit Amsterdam, Department of Clinical, Neuro and Developmental Psychology, Amsterdam Public Health research institute, Amsterdam, Netherlands

##### **Correspondence**: Leonie H. Klaufus (LKlaufus@ggd.amsterdam.nl)

**Objective**

This study aims to validate the Dutch-Flemish PROMIS pediatric item banks v2.0 Anxiety and Depressive Symptoms in a general Dutch population.

**Methods**

Participants (N = 2,893, aged 8 - 18), recruited by two certified internet panel agencies, completed the PROMIS pediatric item banks v2.0 Anxiety and Depressive Symptoms online. Both item banks were assessed on unidimensionality, local dependence, monotonicity, Graded Response Model (GRM) item fit, and differential item functioning (DIF) across gender, age groups, region, ethnicity, and language. The PROMIS pediatric Anxiety and Depressive Symptoms short forms 8a and simulated computerized adaptive testings (CATs) were assessed on reliability and construct validity compared to the Revised Child Anxiety and Depression Scale short version (RCADS-22) subscales.

**Results**

The PROMIS pediatric item banks v2.0 Anxiety and Depressive Symptoms showed sufficient unidimensionality (Omega H = 0.83, 0.95; ECV = 0.79, 0.93, respectively), local independence (residual correlations < 0.2), and monotonicity (H = 0.61, 0.69, respectively). Both item banks showed sufficient GRM item fit (S-X^2^ *p*-value < 0.001), except for the Depressive Symptoms items 2697R1r “I wanted to be by myself“, 7010 “I felt sad for no reason“, and 9001r “I felt too sad to eat”. No DIF was found for gender, age groups, region, ethnicity, and language, except for the Depressive Symptoms items 2697R1r “I wanted to be by myself” and 488R1r “I could not stop feeling sad” that showed uniform DIF for language (McFadden pseudo *R*^*2*^ change > 2%). Based on U.S. parameters, the PROMIS pediatric Anxiety and Depressive Symptoms short forms 8a showed a reliability of > 0.90 in 2% and 34%, and the CATs in 26% and 41% of the participants, respectively. Both short forms and CATs revealed high positive correlations (*r* > 0.70) with the corresponding RCADS-22 subscales and slightly lower correlations with the non-corresponding RCADS-22 subscales (*r* ≤ 0.70).

**Conclusions**

The Dutch-Flemish PROMIS pediatric item banks v2.0 Anxiety and Depressive Symptoms show sufficient psychometric properties, except for four Depressive Symptoms items that show DIF for language or poor GRM item fit; the short forms 8a and CATs seem valid, but reliable for a small percentage of children.

## 132-P. Evaluation of a patient-reported frailty tool in Systemic Lupus Erythematosus

### Sarah B. Lieber^1^, Stephen Paget^1,2^, Jessica R. Berman,^1,2^ Medha Barbhaiya,^1,2^, Lisa R. Sammaritano,^1,2^, Kyriakos Kirou,^1,2^, John A. Carrino,^1,2^, Musarrat Nahid,^2^, Mangala Rajan,^2^, Dina Sheira^1^, Lisa A. Mandl^1,2^

#### ^1^Hospital for Special Surgery, New York, NY; ^2^Weill Cornell Medicine, New York, NY

##### **Correspondence**: Sarah B. Lieber, MD, MS (liebers@hss.edu)

**Objective**

Frailty is associated with disability in systemic lupus erythematosus (SLE). To our knowledge, no phenotypic frailty tool including objective/subjective domains has been compared to a validated point-of-care frailty measure in SLE. We evaluated the point-of-care self-reported FRAIL scale (FS) versus the standard Fried phenotype (FP) by comparing the prevalence of frailty as measured by both tools in a cohort of women with SLE. We also evaluated the association of each frailty measure with several patient-reported outcomes (PROs), comparing associations in frail versus non-frail women.

**Methods**

Adult women <70 years old with validated SLE and mild/moderate disease enrolled from one center. Measures included: frailty (FP/FS); disease activity/damage; and PROs (PRO Measurement Information System (PROMIS) computerized adaptive tests (CATs) and Valued Life Activities (VLA) disability). Differences between frail and non-frail participants were evaluated using Fisher’s exact or Wilcoxon rank sum tests and the association of frailty with disability using logistic regression. Correlation between the FP and the FS was determined using Spearman’s correlation.

**Results**

72 women enrolled; 67 (93%) completed the FS. 17% (FP) and 27% (FS) were frail. Frail women according to either definition had greater disease damage (FP: p=0.002; FS: p=0.0006) and worse PROMIS CATs, including mobility, physical function, pain behavior and interference, and fatigue (FP and FS: all p<0.01). Compared with non-frail women, frail women classified by the FP had greater comorbidity (p=0.02); when classified by the FS, frail women were older (p=0.04) with worse PROMIS CAT depression (p=0.02). Frailty according to either definition was associated with VLA disability after adjustment for age, comorbidity, and disease activity (FP: p=0.02; FS: p=0.0003), but this relationship was attenuated for the FP after adjustment for disease damage (p=0.08). There was moderate correlation between the FS and the FP (r=0.48; p<0.0001).

**Conclusions**

Prevalence of patient-reported frailty was high in this cohort of women with SLE. Frailty, measured with either metric, was associated with worse PROs, providing face validity for both definitions. The FS was associated with disability even after adjustment for multiple confounders. These data suggest that the FS may be an informative point-of-care tool to identify frail women with SLE.

## 133-O. Patient-Reported Outcomes Measurement Information System (PROMIS) - Translation and cultural adaptation of Chinese version of severity of substance use

### Yan Rong, Yun Ting, Shang Meimei, Xu Juan, Huang Ame

#### All authors: Shandong Cancer Hospital and Institute, Shandong First Medical University and Shandong Academy of Medical Sciences)

##### **Correspondence**: Yun Ting (yunting.love@qq.com)

**Introduction**

Patient-reported outcomes and listening to the true feelings of the patient are the hot spot in cancer research both in China and abroad recently. Given the increase in misuse and abuse of prescription opioids, clinicians clearly benefit from a standardized tool to screen screening opioid overuse. In 2009, the International Society for Pharmacoeconomics and Outcome Research (ISPOR), FDA, the Health-related quality of Life working Group and the International Association for quality of Life Research (ISQOL) jointly put forward that incorporating patient self-reporting data into the evaluation system of clinical decision-making, Combined with the patient self-reporting measurement system, it can help clinicians to better detect and screen abnormal drug use behavior, and lay the foundation for early intervention.

**Objectives**

The present study developed a Chinese version of the Severity of Substance Use, and incorporated into the Patient-Reported Outcomes Measurement Information System to promote domestic opioid abuse screening, improve drug evaluation and promote clinical nursing and drug management.

**Methods**

After applying for authorization from the American PROMIS data management center, the translation method of FACIT (Functional Assessment of Chronic Illness Therapy) was adopted. After simultaneous forward translations, reconciliation, back-translation, expert review and proofreading, the first translation draft was formed and submitted to the PNC-China center for quality review. On the basis of the review, cognitive interviews were conducted among 5 cancer patients (at least 5 patients in each item) who were eligible for inclusion, and the interviewees pointed out the items and phrases that were difficult to understand, as well as the possible difficulties in the answer process. The interviews with each patient were recorded and recorded with their consent. The head of the translation team will sort out the patient's feedback, and the cultural mediator will provide the appropriate translation plan with reference to the patient's opinion. After cultural debugging, the final Chinese version of the drug use severity scale was formed.

**Results**

A Chinese version of the severity scale of drug use was formed.

**Conclusion**

We provide a culturally adjusted Chinese version of screening tool for drug abuse in China, and the translation has gone through a standardized process and cultural debugging, which can be used to screen drug abuse in China.

## 134-P. A PROMISing prospect of measuring pediatric general health: A comparison of the PROMIS^®^ pediatric Global Health scale (PGH-7) and the Pediatric Quality of Life Inventory (PedsQL^TM^).

### Michiel A. J. Luijten^1,2^, Lotte Haverman^1^, Raphaële R.L. van Litsenburg^3,4^, Leo D. Roorda^5^, Martha A. Grootenhuis^3^, Caroline B. Terwee^2^

#### ^1^ Emma Children’s Hospital, Amsterdam UMC, University of Amsterdam, Psychosocial Department, Amsterdam, the Netherlands; ^2^ Amsterdam UMC, Vrije Universiteit, Epidemiology and Biostatistics, Amsterdam, the Netherlands; ^3^ Princess Máxima Center for Pediatric Oncology, Utrecht, the Netherlands; ^4^ Emma’s Children’s Hospital, Amsterdam UMC, Vrije Universiteit Amsterdam, ^5^Pediatric Oncology, Cancer Center Amsterdam, Amsterdam, the Netherlands

##### **Correspondence**: Michiel A.J. Luijten (m.a.luijten@amc.nl)

**Objective**

On February 18th 2020 the International Consortium for Health Outcomes Measurement (ICHOM) announced the release of the Standard Set for overall pediatric health. This outcome set contains the Patient-Reported Outcomes Measurement Information System (PROMIS) Pediatric Scale v1.0 Global Health (PGH-7+2) for measuring overall physical, mental and social health. Our aim was to assess the psychometric properties of the PGH-7 in the Dutch population and to compare the performance of the PGH-7 with the Pediatric Quality of Life Inventory (PedsQL^TM^).

**Methods**

Children aged 8-18 years (n=2654), representative of the Dutch population on key demographics were asked to complete the PGH-7 (n_items_=7) and the PedsQL (n_items_=23). To assess structural validity of the PGH-7 a graded response model (GRM) was fitted to the data after assessing the following assumptions: Unidimensionality through CFA (CFI>.95, TLI>.95, RMSEA<.10), local independence by residual correlations (r<.20) and monotonicity by Mokken analysis (*H*>.50, *H*_*i*_>.30). Item fit of the GRM model was inspected with S-X^2^, where *p*<.001 indicates misfit. Additionally, convergent validity of the PGH-7 T-score with the PedsQL total score was assessed. A moderately strong correlation (>.50) was expected, as both instruments measure physical, mental and social domains. Percentage of participants reliably measured was assessed using the standard error of measurement (SEM) <0.32 as a criterion (which equals a reliability of 0.90). Relative efficiency was calculated (1- SEM^2^)/n_items_) to compare how well both instruments perform relative to the amount of items administered.

**Results**

In total 1082 (response rate = 40.8%) children completed both questionnaires. All GRM assumptions were met. PGH-7 displayed good structural (no misfit) and convergent (*r*=.65) validity. Both questionnaires measured reliably (n_PGH-7_=74.5%, n_PedsQL_=76.6%) at the mean and 2SD in clinically relevant direction. The relative efficiency of the PGH-7 was 2.6 in comparison to the PedsQL, indicating that, on average, the items in the PGH-7 are 2.6 times more informative than PedsQL items.

**Conclusions**

The PGH-7 displays sufficient reliability and validity in the general Dutch pediatric population. The scale measures more efficiently than the most commonly used legacy instrument (PedsQL).

## 135-P. How the COVID-19 pandemic impacts the psychosocial well-being of children and adolescents in the Netherlands

### Michiel A.J. Luijten^1,2^, Maud M. van Muilekom^1^, Lorynn Teela^1^, Hedy A. van Oers^1^, Kim J. Oostrom^1^, Lotte Haverman^1^

#### ^1^Amsterdam UMC, University of Amsterdam, Department of Child and Adolescent Psychiatry, Pediatric Psychology and Psychosocial Care, Emma Children’s Hospital, Amsterdam Public Health, Meibergdreef 11, Amsterdam, the Netherlands;^2^Amsterdam UMC, Vrije Universiteit Amsterdam, Department of Epidemiology and Biostatistics, Amsterdam Public Health, De Boelelaan 1117, Amsterdam, the Netherlands

##### **Correspondence:** Michiel A.J. Luijten, MSc; (m.a.luijten@amc.nl)

**Objective**

Recent measures of implementing social isolation and physical distancing as governmental reactions to the COVID-19 outbreak profoundly impact daily life, including that of children and adolescents. Suddenly children and adolescents were not allowed to go to school or participate in sports or other socializing activities anymore. It is therefore relevant to investigate the impact of these measures on psychosocial outcomes in children and adolescents in the general population. In this study we surveyed how the COVID-19 outbreak impacts the psychosocial functioning in a sample of Dutch children and adolescents during the first months of lockdown in one of the largest public health crisis of our time.

**Methods**

In April 2020, children and adolescents aged 8-18 years, representative of the Dutch population on key demographics, were asked to complete the following Patient-Reported Outcomes Measurement Information System (PROMIS^®^) computerized adaptive tests (CATs); anger, anxiety, depressive symptoms, peer relationships, sleep-related impairment and the global health scale, online using the KLIK PROM portal (www.hetklikt.nu). In addition, parents were asked to complete sociodemographic questions about themselves (age, ethnicity, education level) and their child (age, gender, education level and presence of chronic conditions). Finally, both children and parents answered COVID-19 specific questions such as consequences for employment, school and the atmosphere at home. Using independent sample T-tests, PROMIS COVID-19 T-scores were compared to normative data that were collected in the general population pre-COVID (2018; n=1098). Additionally, the same data was gathered simultaneously in a sample of chronically ill children/adolescents and a sample of pediatric psychiatric patients.

**Results and Conclusion**

In total, 902/90/265 children and parents completed all questionnaires for respectively the general population/chronically ill/psychiatric samples. Preliminary results indicate that during the COVID quarantine, children scored significantly (p < 0.001) lower on all domains measured by the PROMIS CATs when compared to pre-quarantine normative data. Children and families experience the quarantine differently, as some children indicate that the atmosphere at home has improved, while others indicate a decline in atmosphere. However, further analyses are required to compare groups on background characteristics and to determine possible relevant covariates that may impact psychosocial functioning. These results will be shown at the conference

## 136-O. Integrating PROMIS CAT collection into Epic: tips for success

### Eric C. Makhni

#### Henry Ford Health System, Detroit, MI USA

##### **Correspondence**: Eric C. Makhni (ericmakhnimd@gmail.com)

**Objective**

There are many significant challenges in implementing PROMIS CAT collection for effective and efficient population health applications. One of the biggest challenges is in effectively integrating this platform with daily clinical operations through the electronic medical record. While third-party platforms offer numerous advantages with regards to customization that may be appealing to medical providers, they can be costly and do not fully integrate into the electronic medical record. The purpose of this presentation is to highlight technical and practical key steps to effectively developing a PROMIS CAT platform within a widely used electronic medical record (Epic, Verona, WI, USA).

**Methods**

A PROM platform was designed with the following objectives: 1) electronic questionnaire assignment fully integrated through the native EHR on a 2) population basis through the orthopedic department, such that all ambulatory patients (and not just surgical patients) received questionnaires. The primary outcome was questionnaire completion rate during an initial pilot implementation. Secondary outcomes included completion rates by questionnaire type, patient age (<45 years, 45-64 years, and 65+ years), and visit type (new or follow-up patient), along with psychometric data of included questionnaires.

**Results**

An automated PROM platform was created through the native workflow and EHR, without the hiring of any additional personnel, utilizing National Institutes of Health (NIH) Patient-Reported Outcomes Measurement Information System (PROMIS) computer adaptive test (CAT) questionnaires. Among the first 1,930 ambulatory encounters and 8,383 questionnaires administered, there was an overall completion rate of 86%, with no questionnaire type completed less than 80% of the time. Questionnaire completion rate among the two youngest age groups (<45 and 45-64 years) was approximately 87%, compared to 83% among patients 65 and older. New patient questionnaire completion rate was 91%, compared to 81% for follow-up patients. There were favorable floor and ceiling effects for all PROMIS questionnaires, with the exception of PROMIS Depression, which had a high floor effect.

**Conclusions**

The results of this pilot study demonstrate feasibility of administering PROMs on a population basis through an EHR. The questionnaire completion rate of (86%) exceeded the target for this pilot phase (60%) and for steady-state implementation (80%). This methodology can serve as a model for effective PROM collection.

## 137-P. Design, development, and implementation of an integrated and automated patient reported outcome measure platform through a native electronic health record: Results from the first 2,000 ambulatory encounters and 8,400 questionnaires administered

### Eric C. Makhni, Jason Davis, Michael Charters, Stephanie Muh, Kelechi Okoroha, Charles S. Day, Theodore Parsons

#### All authors: Henry Ford Health System, Detroit, MI, USA

##### **Corresponding Author:** Eric C. Makhni (ericmakhnimd@gmail.com)

**Background**

Patient reported outcome measures (PROMs) represent the gold standard for reporting patient-centric health state measures in orthopedics. However, routine collection of PROMs in the busy ambulatory setting is challenging due to a number of constraints. The purpose of this study was to design and implement a successful PROM platform through a native electronic health record (EHR).

**Methods**

A PROM platform was designed with the following objectives: 1) electronic questionnaire assignment fully integrated through the native EHR on a 2) population basis through the orthopedic department, such that all ambulatory patients (and not just surgical patients) received questionnaires. The primary outcome was questionnaire completion rate during an initial pilot implementation. Secondary outcomes included completion rates by questionnaire type, patient age (<45 years, 45-64 years, and 65+ years), and visit type (new or follow-up patient), along with psychometric data of included questionnaires.

**Results**

An automated PROM platform was created through the native workflow and EHR, without the hiring of any additional personnel, utilizing National Institutes of Health (NIH) Patient-Reported Outcomes Measurement Information System (PROMIS) computer adaptive test (CAT) questionnaires. Among the first 1,930 ambulatory encounters and 8,383

questionnaires administered, there was an overall completion rate of 86%, with no questionnaire type completed less than 80% of the time. Questionnaire completion rate among the two youngest age groups (<45 and 45-64 years) was approximately 87%, compared to 83% among patients 65 and older. New patient questionnaire completion rate was 91%, compared to 81% for follow-up patients. There were favorable floor and ceiling effects for all PROMIS questionnaires, with the exception of PROMIS Depression, which had a high floor effect.

**Conclusions**

The results of this pilot study demonstrate feasibility of administering PROMs on a population basis using a native electronic health record. The questionnaire completion rate of (86%) exceeded the target for this pilot phase (60%) and for steady-state implementation (80%). This methodology can serve as a model for effective PROM collection.

## 138-P. Role of pre-operative PROMIS scores in predicting post-operative outcomes and likelihood of achieving MCID following arthroscopic rotator cuff repair

### Joseph S. Tramer, Sreten Franovic, Noah Kuhlmann, Colin Schlosser, Alex Pietroski, Vasilios Moutzouros, Stephanie J Muh, Eric C. Makhni

#### All authors: Henry Ford Health System, Detroit, MI, USA

##### **Corresponding Author:** Eric C. Makhni (ericmakhnimd@gmail.com)

**Background**

The Patient-Reported Outcomes Measurement Information System (PROMIS) has emerged as a valid and efficient means of collecting outcomes in patients with rotator cuff tears. The purpose of this study was to examine the role of pre-operative PROMIS computer adaptive test (CAT) scores in predicting post-operative PROMIS CAT scores, as well as likelihood of achieving minimal clinically important difference (MCID) following rotator cuff repair. We hypothesize that pre-operative PROMIS CAT scores will directly impact both post- operative PROMIS CAT scores and likelihood of achieving MCID.

**Methods**

Patients undergoing arthroscopic rotator cuff repair by one of three fellowship-trained surgeons were identified over a 12-month period. Only patients that completed both pre- operative and post-operative PROMIS CAT assessments were included in this cohort. PROMIS CAT forms for upper extremity physical function (PROMIS-UE), pain interference (PROMIS- PI), and depression (PROMIS-D) were utilized. MCID was calculated according to both distribution-based (db) and anchor-based (ab) methodology, and receiver operating characteristics (ROC) were utilized to determine if pre-operative scores were predictive of post- operative outcomes, with 95% specificity.

**Results**

One hundred and seventeen rotator cuff repair patients were included for statistical analysis with surveys completed an average of 29±36 days before and 243±117 days after surgery. PROMIS-UE improved from 30.3 to 38.7 (p<0.05), PROMIS-PI improved from 62.7 to 53.3 (p<0.05), and PROMIS-D improved from 47.4 to 44.3. The average change from pre- operative scores to post operative scores in PROMIS-UE and PROMIS-PI exceeded their dbMCIDs of +3.3 and -2.8, respectively. Similarly, PROMIS-UE, PROMIS-PI, and PROMIS-D exceeded their abMCIDs of 27 +3.1, -4.7, and -3.1, respectively. The percent of patients who met dbMCID for PROMIS-UE, PROMIS-PI and PROMIS-D was 67.8%, 75.4%, and 37.5%, respectively. After introduction of 95% specificity cutoffs, percentage of patients achieving dbMCID for PROMIS-UE, PROMIS PI, and PROMIS-D increased to 86.7%, 88.9%, and 50.0%, respectively. Similarly, the cohort’s probability of achieving abMCID for PROMIS-UE, PROMIS-PI, and PROMIS-D was 66.7%, 64.7%, and 48.2%, respectively. When prognostic cutoffs were introduced, probability of achieving abMCID for PROMIS-UE, PROMIS-PI, and PROMIS-D all increased to 86.7%, 83.3%, and 66.7%, respectively.

**Conclusion**

Arthroscopic rotator cuff repair is an effective surgery for symptomatic patients with rotator cuff tears, resulting in improvements of PROMIS-UE, PROMIS-PI, and PROMIS-D. Pre-operative PROMIS CAT domain scores can be utilized to predict likelihood of achieving or failing to achieve significant improvement across all three health domains.

## 139-P. Presence of preoperative clinical depression does not hinder recovery after anterior cruciate ligament reconstruction

### Eric Guo, Austin Cross, Luke Hessburg, Dylan Koolmes, David Bernstein, Vasilios Moutzouros, Eric C. Makhni

#### All authors**:** Henry Ford Health System, Detroit, MI, USA

##### **Correspondence:** Eric C. Makhni (ericmakhnimd@gmail.com)

**Background**

Current literature suggests a link between psychosocial factors and poor surgical outcomes in patients with musculoskeletal complaints. However, there is a limited body of literature examining the effect of depression on outcomes after anterior cruciate ligament reconstruction (ACLR). The goal of this study is to determine the prevalence of depression in ACLR patients and evaluate its effect on patient-reported outcomes postoperatively.

**Methods**

In this single center retrospective cohort study, 121 pediatric and adult patients who underwent ACLR were included. PROMIS Physical Function (PF), Pain Interference (PI) and Depression (D) scores collected preoperatively and six months postoperatively were reviewed. A PROMIS D ≥ 55 served as a validated threshold for clinical depression. Patients were separated into clinical depression (CD) and no clinical depression (NCD) groups based on preoperative PROMIS D score.

**Results**

121 patients undergoing ACLR were included in this study. 24 (20%) patients met criteria for clinical depression. Preoperatively, the CD group reported lower mean PROMIS PF (34.6 vs. 40.2, [p < 0.01], higher PROMIS PI (65.1 vs. 59.1, [p< 0.01]) than those in the NCD group. Postoperatively, the mean PROMIS PF scores for the CD and NCD group were 48.7 and 51.0, respectively (p = 0.2). Mean postoperative PROMIS PI scores for the CD and NCD cohorts were 52.3 and 48.1, respectively (p = 0.04). After ACLR, there was substantial improvement in PROMIS PF, PROMIS PI in both the CD (+14.1 and -12.8, respectively) and NCD cohorts (+10.8 and -10.4, respectively).

**Conclusion**

Prevalence of preoperative depression in ACLR patients could be as high as 20%. Despite high prevalence of depression preoperatively, there is a significant increase – which exceeds currently accepted MCID values - in PROMIS PF scores after ACLR regardless of presence of preoperative clinical depression. This data suggest that high scores on PROMIS-D pre-operatively do not significantly hinder a patient’s recovery after ACLR.

## 140-P. Establishing and comparing reference pre-operative PROMIS scores in patients undergoing shoulder surgery

### Eric W Guo, Kareeem Elhage, Austin Cross, Luke Hessberg, Caleb Gulledge, Eric C. Makhni

#### All authors: Henry Ford Health System, Detroit, MI, USA

##### **Correspondence:** Eric C. Makhni (ericmakhnimd@gmail.com)

**Background**

The Patient-Reported Outcomes Measurement Information System (PROMIS) has become increasingly popular amongst orthopaedic surgeons treating shoulder pathology. Despite this, there have been few studies that describe and compare preoperative reference scores for specific shoulder surgeries. The primary purpose of this study was to establish and compare baseline preoperative PROMIS scores for three common types of shoulder surgery: rotator cuff repair (RCR), total shoulder arthroplasty (TSA) and labrum repair (LR). The authors hypothesized that PROMIS scores would be sensitive enough such that each surgical group would have a different score compared to the other groups.

**Methods**

In this retrospective cohort study, adult and pediatric patients who underwent surgery for either RCR, TSA, or LR were included. PROMIS-Upper extremity (UE), Pain Interference (PI), and Depression (D) scores that were collected at each patient’s preoperative visit were reviewed. Continuous and categorical variables were compared between operative groups using analysis of variance (ANOVA) and chi-square or Fisher’s exact tests, respectively. Multivariable general linear models were used to identify significant independent predictors of PROMIS scores when controlling for age, sex, and BMI.

**Results**

413 patients were included in the study. 272 were in the RCR group, 84 in the TSA group, and 57 in the LR group. The average LR PROMIS-UE was 39.8 compared to the RCR group (29.9, p < 0.001) and the TSA group (29.6, p < 0.001). There was no difference between the mean RCR and TSA PROMIS-UE (p = 0.93). The average LR PROMIS-PI was 56.6 compared to the RCR group (62.8, p < 0.001) and the TSA group (63.9, p < 0.001). There was no difference between RCR and TSA PROMIS-PI (p = 0.09). The average LR PROMIS-D was 43.5 compared to the RCR group (47.7, p = 0.004) and the TSA group (50.3, p < 0.001). The TSA group also had higher mean PROMIS-D than the RCR group (p = 0.03). For PROMIS-UE and PI, age, BMI, and gender were not found to be significant independent predictors (p = 0.98, 0.88; p = 0.31, 0.48, respectively).

**Conclusion**

Patients undergoing shoulder labrum repair had higher preoperative function scores and lower pain interference and depression scores than those undergoing TSA and RCR. These baseline PROMIS scores should be taken into consideration when tracking a patient’s outcomes after surgery, as a certain score could mean drastically different functional and pain outcomes depending on the underlying pathology.

## 141-P. Withdrawn

## 142-O. Clinically relevant thresholds and meaningful differences for PROMIS Physical Function and I-RODS: patient survey in CIDP

### Rajiv Mallick^1^, Noemi Hahn^2^, Ingemar Merkies^3,4^

#### ^1^CSL Behring, King of Prussia, PA, USA; ^2^Bryter, New York, NY, USA; ^3^Department of Neurology, Maastricht University Medical Center, Maastricht, the Netherlands; ^4^Department of Neurology, St Elisabeth Hospital, Willemstad, Curaçao

##### **Correspondence:** Rajiv Mallick (Rajiv.Mallick@cslbehring.com)

**Objective**

To identify clinically relevant thresholds for PROMIS Physical Function (PF) T- scores and Inflammatory Rasch-built Overall Disability Scale (I-RODS) scores to distinguish disability levels, based on a novel approach in patients with chronic inflammatory demyelinating polyneuropathy (CIDP).

**Methods**

Online global GBS/CIDP Foundation survey data from 426 adults with self- reported CIDP were used to classify two patient-reported outcomes (PROs; PROMIS PF T- scores from the Short Form-4 and I-RODS Rasch-transformed centile scores for social activity/participation) in three disability measures: work impairment, residential changes and need for assistive devices. Chi-square automatic interaction detection (CHAID) was used to identify range of clinically relevant thresholds, meaningful group differences and associated effect sizes (differences/SD) in scores based on most substantial shifts in proportion of patients at highest levels for each disability measure.

**Results**

Mean (SD) PROMIS PF T-score was 36.5 (7.9) (tertiles: 23–33, 34–39, 40–57), and mean I-RODS centile score was 56.2 (16.9). PROMIS PF T-scores of 34–40 (median: 36) and in the highest tertile (41–57; median: 44) were associated with only 5% and 0–2% of patients needing a wheelchair (highest of three levels of need for assistive devices), respectively. By contrast, among those in the highest PROMIS PF T-score tertile, 29% had retired or claimed disability pay (highest level of work impairment) and 15% moved to a single-story home, with family or assisted-living facility (highest level of residential changes). Findings were similar for I-RODS. A group median difference of 6 (23 to 29; effect size: 0.76) in PROMIS PF T-score was associated with greatest shift (39%) in wheelchair dependency from 66% to 27%. By contrast, a group median difference of 11 (23 to 34; effect size: 1.39) was needed to shift highest level of work impairment from 84% to 56%, and a group median difference of 10 (34 to 44; effect size: 1.26) was needed to shift highest level of residential changes from 31% to 15%.

**Conclusions**

Clinically relevant thresholds (range: 36–44) and meaningful differences (range: 6–11; effect size: 0.76–1.39) of PROMIS PF T-scores varied with the underlying cross-sectional anchor (specific disability measure) and physical function trait levels in CIDP patients. Interpretation of meaningfulness of between-group PROMIS PF T-scores may be better informed by realistic assessment of limits in terms of change on an underlying anchor in the context of this high-burden disease.

## 143-P. Development and validation of the Pediatric PROMIS Pain Quality Scale

### A. Mara^1,2^, Adam C. Carle^2,3^, Susmita Kashikar- Zuck^1,2^, Dennis Revicki^4^, Kenneth Goldschneider^2,5^, David D. Sherry^6^, Carlton Dampier^7^, Jennifer Farrell Miller^8^, Kimberly Barnett^9^, Jenna Tress^6^, C. Jeffrey Jacobson^10,11^, Natoshia R. Cunningham^12^, Esi Morgan^2,13^

#### ^1^Behavioral Medicine and Clinical Psychology, Cincinnati Children’s Hospital Medical Center; ^2^Department of Pediatrics, University of Cincinnati College of Medicine; ^3^James M. Anderson Center for Health Systems Excellence, Cincinnati Children's Hospital Medical Center; ^4^Center for Health Outcomes Research, Evidera; ^5^Department of Anesthesiology, Cincinnati Children’s Hospital Medical Center; ^6^Division of Rheumatology, Children’s Hospital of Philadelphia; ^7^Department of Pediatrics, Emory University School of Medicine and AFLAC Cancer and Blood Disorders Center, Children’s Healthcare of Atlanta; ^8^Counseling and Psychological Services, University of Central Florida; ^9^Brigham Young University, Department of Psychology; ^10^Department of Anthropology, University of Cincinnati College of Arts and Sciences; ^11^Family and Community Medicine, University of Cincinnati College of Medicine;^12^Department of Family Medicine, Michigan State University; ^13^Division of Rheumatology, Cincinnati Children’s Hospital Medical Center

##### **Correspondence**: Constance A. Mara (Constance.Mara@cchmc.org)

**Objective**

The primary objective of this study is to evaluate the dimensionality and validity of the Pediatric PROMIS Pain Quality Scale.

**Methods**

The data used in this study included pediatric patients with chronic widespread musculoskeletal pain (fibromyalgia), juvenile idiopathic arthritis, or sickle cell disease ages 8 to 18 treated at three academic medical centers in Ohio, Pennsylvania, and Georgia for a total sample size of N = 447. Initial pools of the pediatric PROMIS pain-related items were developed based on literature reviews, clinician interviews, and qualitative research with patients with chronic pain conditions. Our prior research has focused on the development of three constructs related to pain (pain interference, pain intensity, and pain behavior). The current project focused on the development of a pain quality item bank. A total of 59 candidate items were developed. The pediatric pain quality item bank assesses the specific physical sensations and affective components associated with pain. Because pain can be felt and described in so many ways, this category of pain contains a variety of attributes, such as perceived temperature (e.g., cold), sensations (e.g., throbbing), and perceived affective qualities of pain (e.g., uncomfortable). We conducted confirmatory factor analysis (CFA) to assess dimensionality of the 59 items for pain quality. Of these, 23 items measured "affective" aspects of pain quality and were in the format of “In the past 7 days, did your pain ever feel “(e.g., miserable, unpleasant), with dichotomous response options (yes/no). The remaining items assessed the "sensory" aspect of pain quality and were in the format “In the past 7 days, did your pain ever feel "(e.g., sharp, achy), with a 5-point response option scale ("not at all" to "very much"). Additionally, we developed both sensory and affective pain quality 8-item short forms based on feedback from pain management clinicians.

**Results**

The 59-item unidimensional model fit the data well: comparative fix index (CFI) =.93, Tucker-Lewis index (TLI) =.93, and root mean square error of approximation (RMSEA) = 0.056. The IRT discrimination parameters ranged from 1.05 to 3.81. Three items were excluded due to discrimination parameters less than 1.0. The category threshold parameters for the remaining 56 items ranged from -1.02 to 3.66.

**Conclusions**

The 56-item pediatric PROMIS pain quality item bank includes both "sensory" and "affective" pain quality 8-item short forms that can be used in research and clinical practice. This information may be useful for understanding the condition-specific experiences of pain as well as outcome evaluations.

## 144-O. Agreement between child and caregiver reports across five PROMIS scales in a pediatric burn population

### Kara McMullen^1^, Alyssa Bamer^1^, Lewis Kazis^2^, Cami Rencken^3^, Steven Wolf^4^, Barclay T Stewart^5^, Dagmar Amtmann^1^

#### ^1^University of Washington Department of Rehabilitation Medicine; ^2^Boston University School of Public Health; ^3^Brown University School of Public Health; ^4^University of Texas Medical Branch; ^5^University of Washington Department of Surgery, Harborview Injury Prevention and Research Center

##### **Correspondence**: Kara McMullen (mcmulk@uw.edu)

**Objective**

To examine the agreement between self- and proxy-report on pediatric PROMIS scales of physical function, depression, peer relationships, pain interference, and anger in children and youth who have sustained a burn injury.

**Methods**

Data were collected from children ages 8-17 years who have sustained a moderate to severe burn injury and their caregivers during Burn Model Systems (BMS) National Longitudinal data collection. Self- and proxy-report scales were completed at the same timepoint between 6 months and 15 years after burn injury at regular intervals. The PROMIS-25 and Anger Short Form v1.0 were completed by pediatric burn participants. Caregivers completed either custom (depression, pain) or standard (Physical Function 8a, Peer 7a, Anger 5a) PROMIS proxy short forms. Self- and proxy-report were compared using paired t-tests, effect size (d), Cohen’s weighted Kappa, and intraclass correlation coefficients (ICC(2,1) individual measures). Concordance by levels of severity (≥10 points worse than mean) for each health domain was also examined.

**Results**

A total of 274 child-caregiver pairs completed the PROMIS measures. Mean child age was 13.0 (SD:3) years. Caregivers reported worse scores than the child across all domains, though differences were only significant for physical function, pain interference, and anger (all p≤0.01). Physical function and anger had the largest mean differences at 2.5 and 2.6 points, respectively. Effect sizes ranged from 0.03 (depression) to 0.29 (physical function), with most domains displaying small bias. Kappa values showed moderate to substantial agreement and ranged from 0.52 (pain interference) to 0.69 (depression). Similarly, ICCs were all of moderate agreement and ranged from 0.51 (pain interference) to 0.69 (depression). Concordance rates by severity groups were generally high with 9% (pain) to 19% (peer relationships) of pairs discordant.

**Conclusions**

This study provides support for the use of proxy PROMIS physical function, depression, peer relationships, pain interference, and anger scales in pediatric burn patients. Mean differences between self- and proxy-reports were generally small across all domains and agreement was moderate to substantial. Providers need to be aware that caregivers typically report slightly worse symptoms across all domains compared to child reports.

## 145-P. Enhanced patient reported outcomes to support availability of comprehensive data for telehealth visits during COVID - 19

### Susan Metzger, Kayla Wilbur, Kristina Davis

#### All authors: Northwestern Medicine, Chicago, IL, USA

##### **Correspondence**: Susan Metzger (susan.metzger@nm.org)

**Background**

In the Robert H. Lurie Comprehensive Cancer Center (RHLCCC) of Northwestern Medicine, patient-reported outcome measures (PROMs) integrate into the electronic health record (EHR) to measure patient distress. Patients complete PROMs to screen for anxiety, depression, fatigue, pain, physical function, and supportive care needs. Patients with active electronic patient portal accounts (MyChart) complete the PROMs on a smart phone, tablet, or computer. Some patients (16%) decline or do not activate MyChart. Prior to the COVID-19 pandemic, clinics utilized an iPad or workstation to assist those patients in completion at the time of the visit. This workflow was no longer feasible with increased telehealth visits, leaving patients at high risk of having unidentified distress.

**Objective**

Develop an outreach method to aide patients at RHLCCC of Northwestern Medicine in completing PROMs remotely. Capturing PROMs data is of particular importance to clinicians who are not able to examine the patient during a telehealth visit.

**Methods**

Assisted non-active MyChart patients with upcoming appointments in completing PROMs telephonically during outreach calls. Responses were submitted directly into the EHR. Key workflow components included increasing patient engagement by providing education on PROMs and MyChart and assisting in MyChart enrollment.

**Results**

In total, 869 appointments were identified for outreach calls from March 30 thru June 2020. Throughout the timeframe, 172 (20%) PROMS were completed, 59 (33%) patients agreed to activate their MyChart, and of those who agreed to activate their MyChart, 28 (47%) completed enrollment. To date, 21 (75%) of those who completed enrollment demonstrated continued portal activity, including but not limited to reviewing test results, clinical questions, and additional PROM completion.

**Conclusions**

The COVD-19 pandemic led to a disruption of clinic flow in the RHLCCC of Northwestern Medicine, preventing in person assistance with PROMs completion. Telephone outreach captured data that otherwise would have been missed in elderly and minority patients without means, ability, or access to the electronic MyChart portal. Although too early to measure the impact of telephone outreach on overall PROM completion rates, increase in patient engagement and enrollment on MyChart is vital to the distress screening process.

## 146-P. Is PROMIS a useful outcomes tool for children with Arthrogryposis?

### Kelsey L. Millar^1^, M. Claire Manske^1,2^, Michelle A. James^1,2^

#### ^1^University of California Davis School of Medicine, Sacramento CA;^2^Shriners Hospitals for Children Northern California, Sacramento CA

##### **Correspondence**: Kelsey L. Millar (klmillar@ucdavis.edu)

**Objective**

Arthrogryposis is a potentially disabling congenital condition characterized by contractures of the extremities due to lack of muscle development. Our objective was to determine whether Patient Reported Outcome Measurement Information System (PROMIS) scores would discriminate between children with arthrogryposis and the general pediatric population. We hypothesized that children with arthrogryposis would report impaired Upper Extremity Function and Mobility, but normal Pain Interference and Peer Relationships scores compared to the reference population.

**Methods**

This is a retrospective cohort study of children with arthrogryposis aged 5-17 years who responded to four pediatric PROMIS domain questionnaires (Mobility, Upper Extremity (UE) Function, Pain Interference, and Peer Relationships) during outpatient visits to Shriners Hospital Northern California between April 2017 and May 2019. Responses were converted to a T-score for comparison to a reference pediatric population (mean reference score = 50, standard deviation = 10). For Mobility, UE Function, and Peer Relationships, a T-score ≥ 50 is within normal limits; 40-49 = mild impairment; 30-39 = moderate impairment, and 0-29 = severe impairment. For Pain Interference, a T-score ≤ 49 is within normal limits; 50-59 = mild, 60-69 = moderate, and 70-78 = severe impairment.

**Results**

PROMIS questionnaires were administered to 68 children with arthrogryposis (34 boys, 34 girls) with a mean age of 9.8 years (S.D. 3.8 years). They reported moderately impaired Mobility (38.0±8.9) and UE Function (31.8±12.2), both significantly different than the reference population (p<0.00001). 66% reported moderate or severe impairment with Mobility, and 74% rated their UE Function impairment as moderate or severe. Participants’ Peer Relationship scores were high (54.0±8.3). 97% reported excellent or good Peer Relationships, and 3% reported them as fair. Participants reported normal Pain Interference (49.3±10.0), which was not significantly different than the reference population (p=0.709). 81% reported normal or mild Pain Interference.

**Conclusions**

PROMIS effectively discriminates between children with arthrogryposis and the general population. Children with arthrogryposis report moderately impaired Mobility and UE Function but similar Peer Relationships and Pain Interference in comparison to the reference population. PROMIS is a useful tool to evaluate and understand the challenges that children with arthrogryposis face with respect to their mobility, UE function, peer relationships, and pain interference with activity.

## 147-O. PROMIS Pain Interference scores and Health-Related Quality of Life in patients with end-stage kidney disease

### Istvan Mucsi^1^, Tibyan Ahmed^1,^ Aysha Afzal^1^, Eric Lui^1^, Noshin Ullah^1^, Jessica Li^1^, Susan J. Bartlett^3^, Madeline Li^4^, Doris Howell^5^, John Devin Peipert^6^, Marta Novak^2^

#### ^1^Multi-Organ Transplant Program and Division of Nephrology, University Health Network, Toronto, Canada; ^2^Centre for Mental Health, University Health Network, Toronto, ON, Canada; ^3^Center for Health Outcomes Research, McGill University, Montreal, Quebec, Canada; ^4^Department of Supportive Care, Princess Margaret Hospital, Toronto, Ontario, Canada; ^5^Princess Margaret Cancer Center, Faculty of Nursing, University of Toronto, Toronto, Ontario, Canada; ^6^Department of Medical Social Sciences, Northwestern University Feinberg School of Medicine, Chicago, Illinois

##### **Correspondence:** Istvan Mucsi (Istvan.mucsi@utoronto.ca)

**Objective**

Chronic pain is highly prevalent in patients with end stage kidney disease (ESKD). However, its association with health-related quality of life (HRQoL) among Canadian patients is not fully understood. The US NIH-funded Patient Reported Outcomes Measurement Information System (PROMIS) program has developed and validated tools to assess physical, emotional and social domains across chronic illnesses. The objective of this study was to assess the association of pain interference with HRQoL among patients with ESKD.

**Methods**

Adults with ESKD (dialysis and kidney transplant) completed PROMIS Pain Interference Item Bank, the EuroQOL (EQ-5D-5L) and the SF-12 questionnaires. Sociodemographic and relevant clinical data were collected from medical records. Participants indicated pain interference (exposure), according to the PROMIS T-score metric (range 38-80), with higher score indicating more pain interference. The Canadian valuation set was used to obtain EQ5D5L health utility scores (0-1, 1=best possible health, 0=worst possible health/death). The SF-12 questionnaire, yields a physical component summary (PCS) and mental component summary (MCS) (range 0-100) score, with higher scores indicating better HRQoL.

**Results**

Mean (SD) age (n=523) was 57(17) years. Fifty-seven percent were male, 49% were White (251), 40% were on dialysis, 37% had diabetes. Higher PROMIS pain interference scores were significantly associated with lower HRQoL as measured by the EQ-5D-5L [β= -0.008, 95% [CI]=-0.010, -0.006], p <0.001) after adjusting for age, sex, marital status, education, income, ethnicity, comorbidity, diabetes, renal replacement therapy and additional PROMIS domains such as sleep disturbance and depression. Pain interference was also significantly associated with worse physical (-0.663 [-0.805, -0.521], p<0.001) and mental (-0.184 [-0.291, -0.077], p<0.001) HRQoL in similar multivariable quantile regression models.

**Conclusions**

PROMIS pain interference score was strongly associated with HRQoL. Future research should assess if PROMIS guided screening may improve pain management and HRQoL in patients with ESKD.

## 148-P. PROMIS in clinical practice: Results of qualitative interviews with patients completing patient-reported outcomes

### Therese A. Nelson^1^, Faraz S. Ahmad^1,2^, Martha-Margaret Cotton^2^, Kristina Davis^2^, Leilani Lacson^1^, Ryan Merkow^1,2^, Luke V. Rasmussen^1^, Nan E. Rothrock^1^, Justin B. Starren^1^

#### ^1^Northwestern University, Chicago, Illinois; ^2^Northwestern Medicine, Chicago, Illinois

##### **Correspondence**: Therese Nelson (therese.nelson@northwestern.edu)

**Objective**

Patient-Reported Outcomes (PROs) can elevate the patient voice, but given their more recent introduction into clinical care, it is unclear how patients view PRO questionnaires and why patients often fail to complete them. This presentation will share the patient view of PROs, identified challenges, and a prioritized list of recommendations.

**Methods**

The Electronic Health Record (EHR) Access to Seamless Integration of PROMIS (EASI-PRO) consortium consists of nine universities integrating PROMIS into EHRs. EASI-PRO researchers conducted 23 patient interviews across four clinics at one site. Transcripts were reviewed to examine patient experiences regarding PRO completion, reactions to PRO questions, and physician interaction.

**Results**

Barriers to completion included lack of patient portal access, email overload, confusion between PROs and healthcare satisfaction surveys, challenging physical health, and technical factors. Patients described their experience interpreting email prompts and advised how to make PRO requests more likely to be answered. Patients expressed confusion regarding the purpose of PROs and how they would be used and voiced a desire to learn how results would impact their clinical care. Patients reported that PRO measures themselves were generally understandable but could sometimes be unclear. Their length and content were appropriate. Comments demonstrated the importance of selecting PRO measures that are highly relevant to the patient population, and that completing PRO measures can result in feelings of introspection and gratitude. Patients expressed a strong desire for quick communication of concerning scores and hope that physicians would utilize PRO results to enhance their care. Many patients assumed that the physician would take their PRO results into account and use results to prepare for their medical appointments.

**Conclusions**

In our study, most patients were quite willing to complete PROs, but barriers to completion hampered their response. We will present practical recommendations to address barriers, such as revising the call center script, setting tablets at maximum time-out, communicating expected PRO completion time, informing patients about the purpose of PROs, and refraining from using the word “survey.” Recommendations also focus on patient desires concerning use of PROs in patient care, encouraging clinicians to acknowledge PRO completion and use in the clinical setting.

## 149-P. Premorbid PROMIS® measures and onset of Multiple Sclerosis

### Pamela Newland^1^, Karlie Lading^1^ Ling Chen^2^

#### ^1^Goldfarb School of Nursing at Barnes Jewish College; ^2^Washington University St Louis

##### **Correspondence:** Pamela Newland (Pamela.newland@barnesjewishcollege.edu)

**Objective**

Depression is a common symptom of multiple sclerosis (MS) that has been predicted by a variety of demographic and clinical variables and other symptoms. However, it is unclear if depression is a premorbid symptom prior to diagnosis of MS and its role in clinical decision making. We utilized a large clinical database to enable cross linkage with PROMIS scales and clinical variables of MS.

**Method**

The data network of a large academic center was evaluated to extract PROMIS and other identified variables in both inpatient and outpatients with MS. Keywords were PROMIS, Pain Interference, Anxiety, Depression, and Physical Function with clinical variables of medications for fatigue, year of diagnosis, and diagnosis code for MS (ICD 9 240/ICD 10 G35).

**Results**

Data were available from 260 visits on 66 patients with MS. Patients were predominantly female (61%) and white (90 %) with an average age of 51. PROMIS core item banks were completed by all patients. The year of diagnosis ranged from 1 to 23 years. PROMIS Depression assessment score ranged from 34 to 60 t score.

**Conclusions**

The current work highlights the possible role of premorbid depression as a precursor for disease onset in patients with MS. Additional research is necessary related to the use of PROMIS Depression and other symptom measures in medical decision making for treatment modalities.

## 150-P. Mindfulness art-based therapy - PROMIS Fatigue and influence of Multiple Sclerosis and Global-a pilot study

### Pamela Newland^1^, Karlie Lading^1^, B Ann Bettencourt^2^, Verna Hendricks-Ferguson^3^

#### ^1^Goldfarb School of Nursing at Barnes Jewish College ^2^ University of Missouri Columbia, ^3^Saint Louis University

##### **Correspondence:** Pamela Newland (Pamela.newland@barnesjewishcollege.edu)

**Objective**

Fatigue is a prevalent symptom in patients with multiple sclerosis (MS). Complementary therapies such as mindfulness-based art therapies (MBAT) has potential to minimize fatigue and improve global health. Information is lacking on the patient’s perspective using patient reported outcomes. To determine the patient perspective related to use of mindfulness- based art therapy to improve patient reported outcomes of fatigue and global health.

**Methods**

Community dwelling participants with multiple sclerosis (MS) completed two measures at one time point (i.e., PROMIS Fatigue SF and Global Health were completed). Mean population scoring on each module is defined at 50. Participants also completed a demographic survey that included clinical variables. Bivariate Spearman correlation analysis defined the association between the PROMIS modules and time since diagnosis (in years).

**Results**

Twelve participants with MS took part in the study. All the participants were white, women, average age 48 years, and married, with some college (ranged from 13 to 21 years); and employed. Mean SF and Global scores were similar to values found for MS participants in other studies (39, 30 respectively). The PROMIS Fatigue Scale SF correlated with time since diagnosis (p < .04). There was no correlation between the PROMIS Global LE score and PROMIS Fatigue (p <. 57).

**Conclusions**

The PROMIS Fatigue SF and global health is a useful tool in participants with MS to provide their perspective of symptoms and global health who used MBAT. Further research is needed for follow up the effectiveness of MBAT on fatigue and global health using patient reported outcomes.

## 151-O. Increasing test efficiency in between-item multidimensional computerized adaptive testing by aligning item selection and stopping rules

### Muirne C. S. Paap^1,2^, Johan Braeken^3^

#### ^1^Oslo University Hospital, ^2^University of Groningen, ^3^University of Oslo Abstract

##### **Correspondence**: Muirne C. S. Paap (m.c.s.paap@rug.nl)

**Objective**

Fixed-precision between-item multidimensional computerized adaptive tests (MCATs) are becoming increasingly popular. The current generation of item selection rules used in these types of MCATs typically optimize a single-valued objective criterion for multivariate precision (e.g. Fisher information volume). In contrast, when all dimensions are of interest, the stopping rule is typically defined in terms of a required fixed marginal precision per dimension. This asymmetry between multivariate precision for selection and marginal precision for stopping, has received little attention thus far.

**Methods**

In this presentation, aforementioned selection-stopping asymmetry and its consequences will be discussed, and alternative item selection approaches will be introduced and evaluated. An empirical multidimensional item bank of 194 polytomous items, designed to measure different aspects of quality of life was used as a basis for the simulation study. Four dimensions were measured, using three PROMIS item banks and an additional disease-specific item bank: fatigue (50 items), COPD-specific complaints (46 items), physical function (63 items), and social roles and activities (35 items). The bank was calibrated using a between-item multidimensional graded response model. Higher scores were indicative of higher quality of life for all dimensions. All dimensions were highly positively correlated, and items had high discrimination parameters. The threshold parameters covered a wide range for each dimension.

**Results**

For all but two selection rules, the CAT algorithm reached a proper stop for 100% of the N = 10000 simulees. The longest average total test length was found for the traditional D-rule (12 items), the shortest test length was found for ‘restricted’ and ‘filtered’ item selection rules (7 items); here, items from dimensions for which the required fixed-precision threshold was already met were no longer selected. The traditional rules did not outperform unidimensional CAT in terms of efficiency. For extreme theta values, bias was larger for selection rules that were associated with the shortest tests. Results regarding item usage will be presented as well.

**Conclusions**

Using selection rules which incorporate knowledge on which of the dimensions already meet the required fixed-precision threshold can be expected to result in shorter test lengths for fixed marginal precision MCATs.

## 152-P. Culture in play: spotlighting the universal French translation and linguistic validation of PROMIS item banks

### Emily Parks-Vernizzi^1^, Barbara Perez^1,^ Benjamin Arnold^1^, Abigail Boucher^1^, Helena Correia^3^, Mushirah Hossenbaccus^2^, Sara Ahmed^2†^, Susan Bartlett^2†^

^†^Sara Ahmed and Susan Bartlett are co-senior authors

#### ^1^FACITtrans, Ponte Vedra, FL, USA; ^2^McGill Center for Health Measurement, McGill Medicine, Division of Clinical Epidemiology & MUHC Center for Outcomes Research & Evaluation, Montréal, QC, Canada; ^3^Northwestern University Feinberg School of Medicine, Department of Medical Social Sciences, Evanston, IL, USA

##### **Correspondence:** Emily Parks-Vernizzi, (eparks@facit.org)

**Objective**

The purpose of this study was to translate and linguistically validate 20 PROMIS^®^ adult item banks into Universal French and highlight cultural nuances arising during the translation process.

**Methods**

We translated nearly 600 PROMIS items using the FACIT universal methodology – a standardized iterative process of forward- and back-translation, expert review, harmonization and cognitive interviewing. All members of the translation team were native French-speakers from Belgium, Canada, France, and Switzerland. French-speaking community participants in Canada assessed the relevance, understandability, and appropriateness of the translations. A pragmatic qualitative analyses of cognitive interviews of each translated item was used to identify conceptual and linguistic differences between cultures.

**Results**

The study sample consisted of native French-speaking adults (57 women, 23 men) in Montreal, Canada with a mean age of 37 (20-72). Conceptual and linguistic differences were evident for specific **physical** (“achy” *Pain Quality–Nociceptive*; “bushed” and “wiped out” *Fatigue*; and “do a pull-up” *Physical Function*); **emotional** (“angry” *Depression*); and **social** (“I have trouble” *Ability to Participate*; “people are around me but not with me” *Social Isolation* and “sense of purpose” *Psychosocial Illness Impact – Positive*) items. Interview data revealed that 580 items of the 593 considered items required no revisions. Of the concepts discussed here, only 11 items required iterations to improve conceptual equivalence and two items were revised to accurately reflect the English source.

**Conclusions**

Translating complete PROMIS items banks reveals that while most PROMIS domains are conceptualized and described similarly across cultures, a few items require additional exploration to ensure equivalence. PROMIS universal French item banks in this study are conceptually equivalent to the English source and acceptable for use in international research and clinical trials. Cognitive interviewing in other French-speaking regions is planned. Structured qualitative interviews are essential to assuring the validity of translated items.

## 153-P. Responsiveness of PROMIS short forms among adult cancer patients

### John Devin Peipert^1^, Paul Novotny^2^, Amylou C Dueck^3^, Minji Lee^2,,^ Timothy J Beebe^4,^ Marlene Frost^2^, Kathleen Yost^2^, David Eton^2^, Susan Yount^1^, Jennifer Beaumont^5^, Tito R; Mendoza^6^, Charles S Cleeland^6^, Victoria Blinder^7^, Ethan Basch^8^, Jeff Sloan^2^, David Cella^1^

#### ^1^Northwestern University, Department of Medical Social Sciences; ^2^Mayo Clinic, Rochester, MN; ^3^Mayo Clinic, Scottsdale, AZ; ^4^ University of Minnesota, Minneapolis, MN; ^5^Clinical Outcome Solutions, Los Angeles, CA; ^6^University of Texas M. D. Anderson Cancer Center, Houston, TX; ^7^Memorial Sloan Kettering Cancer Center, New York, NY; ^8^University of North Carolina, Chapel Hill, NC

##### **Correspondence:** John Devin Peipert (john.peipert@northwestern.edu)

**Objective**

The ability of a patient reported outcome measure to reflect changes in health is necessary to support its use in trials and clinical patient monitoring. Though PROMIS measures are commonly used among cancer patients, the responsiveness of some commonly-used PROMIS short forms has not been established among this population.

**Methods**

We used data from a prospective, observational study of 1828 cancer patients. Each participant was surveyed at a baseline timepoint and 6 weeks later on several PROMIS domains, including several short forms of differing lengths within some domains: Physical Function (10a), Anxiety (4a, 6a, 8a), Depression (4a, 6a, 8a), Ability to Participate in Social Roles and Activities (4a, 6a, 8a), Sleep Disturbance (4a, 6a, 8a), Fatigue (7a), Pain Intensity (3a), and Pain Interference (7 item custom short form). Each was scored on a T score metric (mean = 50, SD = 10). We used mixed effects models to estimate the least squares mean change for each short form. Domain specific ratings of change were assessed (e.g., change in physical function over past 6 weeks) and used to categorize change as “better,” “same,” or “worse.” For these groups, we calculated PROMIS change scores. Then we calculated standardized response means (SRM) for each group. SRMs of 0.30 or above were considered evidence of responsiveness.

**Results**

Participants were on average 56 years of age, most often had an ECOG performance status rating of 0 or 1 (71%), and the most common cancer types were breast (26%) and lymphoma/myeloma (21%). Estimated changes in PROMIS scores were most often between 1 and 2.5 T score points. While SRMs for the “better” and “same” change groups were small, those for the “worse” group always exceeded the 0.30 (range: 0.69-0.94). Notably, for domains with multiple short forms, scale length did not affect responsiveness (e.g., Anxiety 4a SRM = 0.69; 6a SRM = 0.69; 8a SRM = 0.70).

**Conclusions**

PROMIS short forms for multiple domains were highly responsive to change in health among a diverse sample of cancer patients. Instruments with more items were not more responsive, indicating the utility of even brief PROMIS assessments.

## 154-O. PROMIS-29 domains associated with dissatisfaction in spine surgery patients who improve in pain and functioning

### Jacquelyn S. Pennings^1,2^, Rogelio A. Coronado^1,2^, Inamullah Khan^1^, Anthony L. Asher^3^, Mohamad Bydon^4^, Clinton J. Devin^5^, Kristin R. Archer^1^

#### ^1^Department of Orthopaedic Surgery, Vanderbilt University Medical Center, Nashville, USA; ^2^Vanderbilt Center for Musculoskeletal Research, Vanderbilt University Medical Center, Nashville, TN; ^3^Carolina Neurosurgery and Spine Associates, Charlotte, USA; ^4^Department of Neurologic Surgery, Mayo Clinic, Rochester, USA; ^5^Steamboat Orthopaedic and Spine Institute, Steamboat Springs, USA

##### **Correspondence**: Jacquelyn S. Pennings (jacquelyn.pennings@vumc.org)

**Objective**

Patient satisfaction is important when evaluating the success of spine surgery. A subset of patients have clinically relevant improvements in disability/pain but report being dissatisfied with surgery. The aim was to evaluate whether changes in mental health (depression and anxiety) and social (ability to participate in social roles [SR]) domains of the PROMIS-29 were associated with dissatisfaction at 1-year after spinal surgery for patients who achieve clinical improvement in disability or extremity pain.

**Methods**

The study was a retrospective analysis of data collected between 2018-2019 from a prospective spine surgery registry, Quality Outcomes Database. Participants completed the PROMIS-29, ODI, and back/leg NRS pain ratings preoperatively and 1-year after surgery. Patient demographic and clinical characteristics were collected from medical records and patient interviews. Satisfaction was assessed at 1-year with 1-item from the NASS lumbar spine outcome assessment. Participants (N=369) undergoing elective surgery for degenerative spine conditions and having a successful outcome (achieving at least 30% improvement in disability or leg pain) 1- year after surgery were included. Logistic regression predicted dissatisfaction at 1-year from PROMIS-29 domain T-scores (SR, anxiety, depression) at 12 months. Covariates included demographic, clinical, surgical characteristics, preoperative PROMIS scores and disability, and postoperative complications and revision surgery after surgery.

**Results**

A total of 116 participants (31%) with clinical improvement in disability or leg pain reported being dissatisfied at 1-year after surgery. When controlling for baseline scores, ability to participate in social roles (OR=0.87, 95%CI=0.84-0.90, p<0.001), depression (OR=1.09, 95%CI=1.06-1.13, p<0.001), and anxiety (OR=1.05, 95%CI=1.02-1.07, p<0.001) at 12 months were all significantly associated with dissatisfaction at 1-year post surgery. None of the preoperative PROMIS domains were associated with dissatisfaction at 1-year (p < 0.05).

**Conclusions**

12-month PROMIS scores were significantly associated with dissatisfaction indicating that patients with who had less improvement in social activities, depression, and anxiety tended to report being dissatisfied even after having a clinically relevant improvement in disability/pain. Preoperatively, none of these PROMIS scores were associated with dissatisfaction at 1-year. The results indicate that improvements in social and mental health factors play a role in patient satisfaction after lumbar spine surgery along with improvements in disability and pain.

## 155-O. Withdrawn

## 156-P. Patient reported outcomes after risk-reducing gynecologic surgery for hereditary breast and ovarian cancer syndromes

### Lauren Philp^1^, Stephanie Alimena^2^, Mackenzie Sullivan^2^, Whitfield B Growdon^1^, Amy J Bregar^1^, Thomas Randall^1^, Katelyn Dorney^1^, Annekathryn Goodman^1^, Eric Eisenhauer^1^, Marcela del Carmen^1^, Rachel Clark Sisodia^1^

#### ^1^Department of Obstetrics and Gynecology, Division of Gynecologic Oncology, Massachusetts General Hospital, Boston, MA; ^2^Department of Obstetrics and Gynecology, Massachusetts General Hospital and Brigham and Women’s Hospital, Boston, MA

##### **Correspondence:** Lauren Philp (lphilp@mgh.harvard.edu)

**Objective**

To determine the impact of risk-reducing gynecologic surgery (RRGS) on the health-care quality of life (QOL) of women with hereditary breast and ovarian cancer syndromes (HBOCS) and to compare these outcomes to patients with benign and malignant ovarian disease.

**Methods**

Patient reported outcome (PRO) collection was implemented at our gynecologic oncology clinic in January 2018. At serial visits, patients were administered general and disease specific PRO measures (PROMs) based on patient disease site. Cohorts of patients with ovarian cancer (OC), HBOCS or benign ovarian masses (BOM) were identified and additional clinical and surgical characteristics were collected prospectively. Specific PROM questions reflecting important physical and psychosocial outcomes were selected a-priori from questionnaires for analysis. Over the study period, both first and last and pre- and post-operative PROM responses were described and compared between cohorts.

**Outcomes**

Between January 2018 and October 2019, 150 HBOCS patients, 209 BOM patients and 329 OC patients were identified. In the HBOCS cohort, PROM responders were similar to non- responders, however, were significantly younger than OC responders (p<0.001). During the study period, 24.7% of HBOCS patients had RRGS. Post-operatively, patients reported feeling less tense (p=0.034) and less worried about future health (p=0.020) but did report more difficulty sleeping (p=0.011), less interest in sex (p=0.025) but no changes in body image. Patients did not report feeling burdened by their treatment. When first and last PROM responses were compared in the HBOCS patients who did not have RRGS, no significant changes were noted. When first PROM responses were compared between cohorts, HBOCS and BOM patients were similar however HBOCS patients reported better QOL (p=0.015) and overall health (p=0.008).OC patients reported the worst QOL (p=0.008), highest levels of worry (p=0.048) and treatment burden (p<0.001), lowest overall health (p=0.003) and highest disease interference in their family life (p=0.003), social life (p<0.001) and finances (p=0.008). When last PROM responses were compared between groups, a similar trend was noted.

**Conclusion**

Patients with HBOCS report overall good QOL after RRGS and better QOL than patients with ovarian malignancies. These results can help to guide counselling for patients with HBOCS and to address their unique health-care needs.

## 157-P. Validation after translation of PROMIS-57 Profile Norwegian with factor analysis, IRT and DIF analysis

### Stein Arne Rimehaug^1^, Aaron James Kaat^2^, Jan Egil Nordvik^3^

#### ^1^University of Oslo, Norway, ^2^Nortwestern University, Chicago IL; ^3^Sunnaas hospital, Oslo

##### **Correspondence:** Stein Arne Rimehaug (stein.arne.rimehaug@sunnaas.no)

**Objective**

Cross-sectional reliability and validation after translation of seven PROMIS**®** Short forms in a Norwegian general population, n=408.

**Methods**

Anonymous, voluntary online collection of demographics, RAND36 and PROMIS57 (including 8-item short forms for physical function, anxiety, depression, fatigue, sleep disturbance, ability to participate in social roles and activities, and pain interference). Analysis: Correlations against similar/dissimilar domains in PROMIS57 and RAND36, bi-factor, EFA, CFA and Mokken analysis checking factor structure and IRT assumptions. IRT Graded Response model: Item and model fit, ICC, TIF and SE plots. DIF analysis in lordif (R) with ChiSquare and McFadden R2 methods for language DIF against Wave1 and ProfilesHUI datasets, and demographic DIF.

**Results**

Reliability>.9, concurrent validity correlations .7-.9 and discriminant correlations .4-.7. CFA 7-factor with WLSMV estimator results in scaled model fit indices of: RMSEA=.05, CFI=.99, TLI =.99 supporting the structural validity. First factor eigenvalue ratios from bi-factor analysis between 4:1 (Sleep) and 32:1 (Social), and Estimated Common Variance (ECV) per domain between 86 and 96 (>60 supports unidimensionality). Local Dependence: 4 or less out of 196 possible item pairs flagged with CFA residuals >.2, or IRT Chen&Thissen LD index >.3. Only two misfitting items (Sleep 44 and 72), based on s-x2. Graded Response model fit: RMSEA=.13, SRMSR: .14, TLI and CFI: .96., and acceptable IRT plots. Scores in this general population sample are skewed and zero-inflated. Sample: 74% women, mean age 52.

**Conclusions**

For each domain (=short form) excellent reliability, and concurrent and discriminant validity. Factor structure of PROMIS 57 seven domains confirmed. IRT assumptions are met for unidimensionality, local independence, monotonicity and invariance (=no language DIF, age, gender or education DIF). Issues: very high discrimination parameters may be related to skewed/zero-inflated distribution, sub-threshold LD/dimensionality issues, and sample size. Lessons learned: obtain a larger and more diverse sample for IRT and DIF. Translate and assess entire item banks at once, rather than profiles and short forms.

## 158-P. Word selection for translating PROMIS® Fatigue items, using a lay person panel

### Stein Arne Rimehaug stein.arne.rimehaug@sunnaas.no

#### University of Oslo and Sunnaas Rehabilitation Hospital, Norway

**Objective**

To systematically translate words indicating varying degrees of fatigue in the PROMIS Item bank with the help of lay person input.

**Methods**

Comparing the rank order of available words expressing different degrees of fatigue in Norwegian by asking a small cognitive debriefing panel, n=5, to rate each expression on a 10-point NRS scale. The rank order and standard deviation for each expression helps indicate which words should match the severity of each English language counterpart in the PROMIS Fatigue Item Bank.

**Results**

14 words/expressions tested. Agreement among blinded participant, median SD 1.3 on a 10point scale. Useful supplement to expert and translator opinion. The resulting ranking could not be used directly for word selection, as the semantic meaning not always matched. Also some words are already translated by FACITrans in FACT or NeuroQol items.

**Conclusions**

Many PROMIS fatigue items hinge the severity onto a single word or expression, to a greater degree than other short forms and item banks. While translating the Fatigue short form, we wished to avoid making word choices that would “use up” words that woulfd be a better fit later for other items in the bank. This ranking by a panel helped inform the process, though the panel perhaps should have had more respondents.

## 159-P. PROMIS sleep disturbance and sleep-related impairment item banks in the Dutch general population

### CB Terwee^1^, M Avetisyan^1^, MHP Crins^2^, LD Roorda^2^

#### ^1^Amsterdam UMC, Vrije Universiteit Amsterdam, Department of Epidemiology and Biostatistics, Amsterdam Public Health Research Institute, Boelelaan 1117, Amsterdam, the Netherlands; ^2^Amsterdam Rehabilitation Research Center | Reade, Amsterdam, the Netherlands;

##### **Correspondence**: Leo Roorda, (leo.d.roorda.research@gmail.com)

**Objective**

The Patient-Reported Outcomes Measurement Information System (PROMIS®) v1.0 item banks ‘Sleep Disturbance’ (SD, 27 items) and ‘Sleep-Related Impairment’ (SRI, 16 items) were developed to measure self-reported aspects of sleepiness, sleep quality, and functional impact of sleep problems more efficiently and precisely than current instruments, by using Computerized Adaptive Testing (CAT). We validated these item banks in a Dutch general population.

**Methods**

Participants in an internet panel completed both item banks. Unidimensionality, local dependence, monotonicity, Graded Response Model (GRM) item fit, Differential Item Functioning (DIF) for age, gender, education, region, ethnicity, and language (Dutch compared to US Wave 1 data), and reliability were assessed.

**Results**

A representative Dutch sample of 1006 people participated. We found sufficient unidimensionality for the both item banks (SD: CFI=0.93, TLI=0.92, RMSEA=0.13, Omega H=0.80, ECV=0.69; SRI: CFI=0.96, TLI=0.95, RMSEA=0.17, Omega H=0.85, ECV=0.76). Some local dependence was found (SD: 4.8%; SRI: 0.8% item-pair correlations>0.20), sufficient monotonicity (SD: H=0.60; SRI: H=0.65), and good IRT item fit (SD: zero out of 27 items with S-X^2^ p-value <0.001; SRI: two out of 16 items). For SD DIF for age was found for four items; younger persons report more sleep problems compared to older persons with similar levels of sleep disturbances. No DIF was found for SRI. We found a reliability of at least 0.90 with simulated CATs (based on US item parameters) in 96% of the participants with on average 4.5 (range 2-12) items for SD and 75% of the participants with on average 6.2 (range 3-12) items for SRI.

**Conclusion**

The PROMIS sleep item banks showed sufficient psychometric properties in a general Dutch population and can be used as CAT. PROMIS CATs allow reliable and valid measurement in an efficient and user-friendly way with limited administration time.

## 160-O. Reducing patient and provider burden: methodology for automated collection of PROMIS CAT in EPIC

### Stacy Schmitt^1^, Martha Springsted^1^, William Mauck, MD^2^, Andrea Cheville^3^, Timothy Maus^4^

#### ^1^Mayo clinic Rochester, MN Multidisciplinary Spine Center; ^2^Departments of Anesthesiology; _3_Physical Medicine; ^4^Rehabilitation, and Radiology

##### **Correspondence**: Stacy Schmitt (Schmitt.Stacy@mayo.edu)

**Objective**

Stakeholder burden is an obstacle to patient reported outcomes (PROs) data collection across the continuum of spine care. The objective is to describe methods to automate administration of 8 PROMIS domains via computer adaptive testing (CAT) within the Epic electronic health record (EHR) throughout a high volume, multisite regional spine care practice to improve patient response rates and unbiased outcome assessment.

**Methods**

Collaborative efforts between 11 Mayo spine care provider specialties (representing over 1,000 providers), project managers, and EPIC data architects provided the architect team an understanding of clinical and research requirements for data collection. Iterative builds and testing of code ensued.

**Results**

Consensus was achieved among all spine care providers to move from legacy instruments to 8 PROMIS domains assessed via CAT. Anchor events (EPIC defined visit types, procedure and surgical codes) were used as triggers to initiate a predefined cadence (baseline, 3, 6, 12, 24…60 months) of PROMIS CAT assignments. EHR logic was developed to automatically cease data collection and re-initiate the baseline and cadence collection as the patient traversed diverse spine care trajectories potentially including primary care, spine specialty care, interventional pain procedures, and surgery. PRO collection modes include: electronic patient portal > on-site tablet > interactive voice response > manual phone contacts. Epic reports were parameterized to assess patient response rates on a clinical site-specific basis for quality assurance and to direct efforts to improve response rates. An Epic registry and dashboard were created to display longitudinal patient-level and aggregated data. Epic’s registry function integrated clinical, laboratory, imaging and surgical data with the PROMIS data. Artificial intelligence and conventional analytic efforts are planned to identify and validate patient phenotypes that predict care trajectories and may be used to inform shared and clinical decision making.

**Conclusions**

A system was created in the Epic EHR for automated CAT assessment of PROMIS domains in order to measure the effectiveness of intensity and sequence of spine care in a quaternary, regional health system. Patient response rates distinguished by site of care, mechanism of data collection, and follow up interval will be presented, along with Epic display and analysis tools.

## 161-O. Incorporating PROMIS into the CIBMTR hematopoietic cell transplant outcomes registry

### Bronwen E Shaw^1^, Deborah Mattila^2^, Linda J Burns^1^, Ruta Brazauskas^1^, Erin Leckrone^2^, Rachel Cusatis^1^, Kathryn E Flynn^1^

#### ^1^CIBMTR, Froedtert and the Medical College of Wisconsin, Milwaukee, USA; ^2^CIBMTR, NMDP/BetheMatch, Minneapolis, USA

##### **Correspondence**: Bronwen Shaw (beshaw@mcw.edu)

**Objectives**

Hematopoietic cell transplantation (HCT) is an established therapy to treat many hematologic diseases. The Center for International Blood and Marrow Transplant Research (CIBMTR) is an outcome registry that has been collecting clinical outcome data for HCT recipients for over 45 years. There are >540,000 unique patients represented in the registry with longitudinal data collected, including demographics, exposures, clinical outcomes and late effects, linked to bio- repository samples. Important clinical questions using this CIBMTR data are proposed by the community and facilitated by CIBMTR scientific and statistical experts. However, to date there is no routine collection of Patient-Reported Outcomes (PROs). Our objective was to incorporate routine PRO collection into the CIBMTR registry.

**Methods**

CIBMTR performed two studies to assess the feasibility of centralized PRO collection. The first used pen-and-paper methodology and local consenting, the second used a bespoke electronic (ePRO) system through which consent was obtained by CIBMTR. Both required local site Institutional Review Board (IRB) approval (in the second for sites to provide patient contact details to CIBMTR). The ePRO system incorporates a patient-friendly interface in Qualtrics, an API link to the PROMIS measures delivered using CAT technology, links to a contact management system to track and trigger PROs, and links to the CIBMTR database to store PROs and link them to the clinical data.

**Results**

The first study confirmed feasibility and acceptability of centralized PRO data collection, but highlighted barriers imposed by pen-and-paper methodology. The second study showed successful implementation of the ePRO system with logistic efficiencies, ease of electronic consenting and PROMIS data collection (with an average of 4.2-7.4 questions completed in 0.7- 1.2 minutes per domain), and successful linkage with clinical data, but delays related to local IRB approvals. To address this CIBMTR developed a mechanism to collect patient contact details and operate the PRO collection under a single centrally IRB-approved protocol.

**Conclusions**

Using these strategies, CIBMTR can now incorporate routine PROMIS PROs for HCT recipients whose clinical data is included in the registry. This has enormous potential for future uses of these data to answer important research questions in a real-world data setting.

## 162-P. PROMIS scores for children with Unilateral Congenital Below Elbow Deficiency (UCBED)

### Azarin Shoghi^1^, Anita Bagley^1,2^, Michelle A. James^1,2^

#### ^1^Shriners Hospital for Children, Northern California (SHCNC); ^2^University of California, Davis School of Medicine; Sacramento, CA, USA

##### **Correspondence:** Michelle James (MJames@shrinenet.org)

**Objective**

Children with UCBED are congenitally one-handed. In previous studies^(1,2)^, they have milder- than-expected disability. Although PROMIS discerns UE function in other congenital arm differences^(3)^, results for children with UCBED are not known. This study evaluates their responses to PROMIS Short Form Upper Extremity Function (UEF), Mobility, and Peer Relationships (PR) domains.

**Methods**

This is a retrospective review of children with UCBED who responded to PROMIS questions from 4/1/17-3/31/20, by parent-proxy (5-7 years) or self-report (8-17). Responses were converted to a T-score for comparison to a reference population. T-score≥50=normal; 40- 49=mild, 30-39=moderate, and 0-29=severe impairment.

**Results**

28 children/proxies completed questionnaires (mean 11±3.4 years). They scored the same as the reference population for Mobility and PR, and reported lower UEF (43.5±9.3; P=0.02). 5-7 year-olds (5 children) reported normal PR, mildly impaired Mobility (46.0 ± 5.6), and moderately impaired UEF (30.2 ± 3.5; P=0.003); 8-12 year-olds (13) reported normal PR and Mobility and moderately impaired UEF (42.7±8.0). 13-17 year-olds (10) reported normal outcomes on all three domains.

**Conclusions**

1. Impairment measured by PROMIS is similar to previous studies for children with UCBED.

2. 5-7 year-olds reported lower UEF scores; short Form UEF tasks may be too difficult, and/or parents may under-report function (4). A study of typically developing 5-7 year- olds is underway to determine whether the UEF Short Form questions have a floor effect for this age group.

3. PROMIS UEF may have a ceiling effect for older children with UCBED.

4. Children with UCBED have a potentially stigmatizing UE difference, but do not report challenges with peer relationships.

**References**

(1) James MA et al. Impact of prostheses on function and quality of life for children with unilateral congenital below-the-elbow deficiency. JBJS 2006; 88:2356.

(2) Bagley AM et al. The Unilateral Below Elbow Test: a function test for children with unilateral congenital below elbow deficiency. Dev Med Child Neuro 2006; 48:569.

(3) Oishi S et al. Treatment and outcomes of arthrogryposis in the upper extremity. Am J Med Genet 2019.

(4) Sheffler LC et al. Comparison of self-reports and parent proxy-reports of function and quality of life of children with below-the-elbow deficiency. JBJS 2009; 91:2852.

## 163-P. Measurement properties of PROMIS short forms for pain and function in three samples of orthopaedic patients

### Anika Stephan^1^, Vincent Stadelmann^1^, Michael Leunig^2^, Franco Impellizzeri^3^

#### ^1^Department of Teaching, Research and Development, Schulthess Clinic, Zurich, Switzerland; ^2^Department of Hip and Knee Surgery, Schulthess Clinic; ^3^Faculty of Health, University of Technology Sydney

##### **Correspondence:** Anika Stephan, (anika.stephan@kws.ch)

**Objective**

The aim of this study was to evaluate the measurement properties of the German PROMIS- short forms (SF) for pain intensity 3a (PAIN), pain interference 4a (PI) and physical function 4a (PF) in patients undergoing total knee arthroplasty (TKA), total hip arthroplasty (THA) or foot/ankle surgery (F/A).

**Methods**

PROMIS-SF data were taken from our clinic registries for the respective patient groups pre-, 6 and 12 months post-surgery (THA, TKA only). Higher PROMIS-SF scores indicate more PAIN, higher PI and better PF. Main reference measures were the Oxford Knee Score (OKS), Oxford Hip Score (OHS) and Foot Function Index (FFI-D). A subsample completed pre-surgery or 6-month PROMIS-SF twice within 14 days to test reliability.

**Results**

Baseline (and longitudinal) sample sizes were: TKA, 144 (120); THA, 132 (116); F/A, 748 (202). Test-retest sample sizes ranged from 45 to 65. Correlations with reference measures were r≥0.7 for TKA and THA, and r≥0.6 for F/A. Cronbach’s α indicated appropriate internal consistency for all SF [0.84≤α≤0.93] in all groups. Intraclass correlation coefficients were best for TKA (0.9-0.97), good for F/A (0.81-0.91) and acceptable for THA (0.69-0.81). Standard errors of measurement represented the following percentages of the mean score change: TKA, 14-23%; THA, 16-21%; F/A, 30-60%. Smallest detectable change thresholds (SDC90) were: PAIN, 7 points (all groups); PI, 7-8 points (all groups); PF, 8-9 points (THA, F/A); PF, 4 points (TKA). Minimal important changes could be calculated for TKA and F/A, and were around 8-9 and 4-5 points, respectively. All three groups showed follow-up ceiling effects (best score) in the PF scale: TKA, 30%; THA, 66%; F/A, 41%. Correlations of PROMIS change scores with the main reference instruments’ change scores were good for TKA [0.52≤│r│≤0.65] and THA [0.73 ≤│r│≤0.8], but limited for F/A [0.42≤│r│≤0.56].

**Conclusions**

PROMIS-SF of pain and function could be used in orthopaedic patients. However, the standard error of measurement showed one to two thirds of the mean change for F/A patients, which limits the interpretation of change. Furthermore, improvement of PF in THA patients might be underestimated, as the follow-up PF score showed large ceiling effects.

## 164-P. Predicting scores for the EORTC QLQ C-30 using linear modeling of PROMIS Global Health responses

### Yujie Sun^1^, Robert Feldman^2^, Andrew Althouse^2^, Dhiraj Yadav^3^, Anna Evans Phillips^3^

#### ^1^Department of Medicine, University of Pittsburgh School of Medicine, Pittsburgh, PA, USA; ^2^Center for Research on Health Care Data, Department of Medicine, University of Pittsburgh School of Medicine, Pittsburgh, PA, USA; ^3^Division of Gastroenterology, Hepatology and Nutrition, Department of Medicine, University of Pittsburgh School of Medicine, Pittsburgh, PA, USA

##### **Correspondence**: Yujie Sun (Suny4@upmc.edu)

**Background**

Translation of data between patient reported outcome (PRO) tools allows for pooling and comparison of data between similar patient populations. To date there exists no established method for prediction of European Organization for the Research and Treatment of Cancer Quality of Life Questionnaire (EORTC QLQ C-30) scores from the Patient Reported Outcomes Measurement Information System Global Health questionnaire (PROMIS GH). This study’s aim was to create a prediction method for the EORTC QLQ C-30 based on PROMIS GH responses.

**Methods**

The EORTC QLQ C-30 (Version 3.0) and PROMIS GH (Version 1.2) were administered prospectively at the University of Pittsburgh Medical Center to self-described healthy subjects who were control volunteers for a study on pancreatic pain. These subjects had neither pancreatic disease or abdominal pain. Multivariable regression models were completed with EORTC QLQ C-30 subscores (Quality of Life (QOL), physical, role, emotional, cognitive, and social functioning) as dependent variables and the PROMIS items as independent variables. Adjusted R^2^ and model p-value were reported for EORTC QLQ C-30 subscales.

**Results**

A total of 220 subjects (Mean age 43.8 ± 18 years, males n= 90 (41%)) were analyzed. Mean composite PROMIS Mental Health T-score was 53.68 ± 9.08, and composite Physical Health T- score was 55.68 ± 7.45, confirming healthy status of the population. Range of mean scaled composite scores for EORTC QLQ-C30 was 84.2 ± 15.79 to 95.83 ± 12.6. EORTC QLQ C-30 QOL score showed the highest correlation with between actual and predicted values (adjusted R^2^=0.638; p<0.001). The emotional functioning subscore also showed close correlation between observed and predicted values (adjusted R^2^=0.623; p<0.001). Modest to poor correlation was seen for physical (adjusted R^2^=0.480), social (adjusted R^2^=0.372), role (adjusted R^2^=0.292), and cognitive functioning scores (adjusted R^2^=0.289; all p<0.001). Higher correlations between actual and predicted values were seen with items containing direct content overlap between the two PRO tools.

**Conclusion**

PROMIS-GH can be used to predict EORTC QLQ C-30 QOL and emotional functioning subscore values using linear regression modeling. Additional subscores cannot be predicted with more than moderate correlation to actual scores due to lack of content overlap between the PRO tools.

## 165-P. Common patient-reported outcomes across ICHOM standard sets – The value of PROMIS^®^

### Caroline B Terwee^1^, Marloes Zuidgeest^2^, Harold E Vonkeman^3^, David Cella^4^, Lotte Haverman^5^, Leo D Roorda^6^

#### ^1^Amsterdam UMC, Vrije Universiteit Amsterdam, Department of Epidemiology and Biostatistics, Amsterdam Public Health Research Institute, Boelelaan 1117, Amsterdam, the Netherlands; ^2^National Health Care Institute, Willem Dodukhof 1, Diemen-Zuid, the Netherlands; ^3^Department of Rheumatology and Clinical Immunology, Medisch Spectrum Twente and University of Twente, Enschede, the Netherlands; ^4^Department of Medical Social Sciences, Northwestern University Feinberg School of Medicine, Chicago, US; ^5^Emma Children’s Hospital Amsterdam UMC, University of Amsterdam, Psychosocial Department, Meibergdreef 9, Amsterdam, The Netherlands; ^6^Amsterdam Rehabilitation Research Center | Reade, Amsterdam, the Netherlands

##### **Correspondence:** Caroline Terwee (cb.terwee@amsterdamumc.nl)

**Objective**

The International Consortium for Health Outcomes Measurement (ICHOM) develops condition-specific Standard Sets of outcomes to be measured in clinical practice for value-based healthcare evaluation. There are, however, large differences and inconsistencies between sets in selected patient-reported outcomes (PROs), terms and definitions used, and recommended patient- reported outcome measures (PROMs), even for the same PROs, which threatens the validity and practical applicability of the ICHOM Standard Sets. It would be ideal if common PROs would be named and defined similarly and measured with the same PROMs across conditions. PROMIS® offers an evidence-based conceptual framework of commonly relevant PROs and validated PROMs that are applicable across patient populations and medical specialties. The aim of this study was to identify shared PROs across ICHOM Standard Sets and to examine to what extend these PROs can be measured with PROMIS.

**Methods**

All individuals PROs and recommended PROMs were extracted from all available ICHOM Standard Sets in January 2020. Similar PROs were categorized into unique PRO concepts. Subsequently, it was examined which of these PRO domains can be measured with PROMIS.

**Results**

In 28 ICHOM Standard Sets, 182 PROs were identified. A total of 96 different PROMs are recommended for measuring these PROs. The 182 PROs were categorized into 21 unique PRO concepts. More than half (12/21) of these PRO concepts (covering 74% of the 182 PROs and 79% of the 96 PROMs) can be measured with a PROMIS measure. Furthermore, inconsistencies were found in the selected PROs and PROMs across Standard Sets. It is unclear why some PROs are included in some Standard Sets, but not in others.

**Conclusion**

Considerable overlap was found in PROs across ICHOM Standard Sets, and large differences in terms used and recommended PROMs, even for the same PROs. Inconsistencies in the selected PROs and PROMs across Standard Sets questions the validity of the Standard Sets. We recommend a more universal and standardized approach to PRO and PROM selection, using a common measurement system such as PROMIS, to improve the validity of outcome measurements in clinical practice, and facilitate benchmarking, learning and improve quality of care across patient groups.

## 166-P. Smallest Detectable Change (SDC) and Minimal Important Change (MIC) of PROMIS instruments – A systematic review

### CB Terwee^1^, JD Peipert^2^, R Chapman^2^, P Griffiths^3^, LB Mokkink^1^

#### ^1^Amsterdam UMC, Vrije Universiteit Amsterdam, Department of Epidemiology and Biostatistics, Amsterdam Public Health Research Institute, Boelelaan 1117, Amsterdam, the Netherlands; ^2^Department of Medical Social Sciences, Feinberg School of Medicine, Northwestern University, Chicago, USA; ^3^ Adelphi Mill, Bollington, Cheshire, UK

##### **Correspondence:** Caroline Terwee (cb.terwee@amsterdamumc.nl)

**Objective**

To summarize available evidence on Smallest Detectable Change (SDC, smallest change in score that is not due to measurement error) and Minimal Important Change (MIC, smallest change that patients, on average, consider important) of PROMIS measures and provide method recommendations.

**Methods**

A systematic PubMed search was performed to identify all studies that evaluated test- retest reliability or estimated a MIC value of any PROMIS measure in any population. The quality of reliability studies was assessed with the COSMIN Risk of Bias checklist and SDC was extracted or calculated from test-retest reliability standard error of measurement or limits of agreement. Anchor- based MIC values were extracted (which are preferred over distribution-based methods).

**Results**

Twenty-five studies examined test-retest reliability. Only five studies provided evidence on SDC, of which three were rated as doubtful or adequate quality. These three studies reported SDC values between 7.7-16.3 T-score points for 16 PROMIS measures. Twenty-two studies evaluated the MIC of one or more PROMIS measures, of which 16 used anchor-based methods. MIC was most often defined as a mean change in PROMIS T-score in patients who slightly improved on an anchor. Anchors, however, did not always measure the same construct as the PROMIS measure and sample sizes were often small. Most MIC values were found for adult Pain Interference (14 studies, MIC values 0.7-12.4), Physical Function (13 studies, MIC values 0.1-12.0), Anxiety (6 studies, MIC values 0.2-8.0), Depression (5 studies, MIC values 2.1-5.8), Fatigue (5 studies, MIC values 1.5-7.6), Satisfaction with Social Roles and Activities (4 studies, MIC values 0.6-6.2), and Sleep Disturbance (3 studies, MIC values 1.2-6.6). Only two studies estimated MIC values for pediatric item banks, MIC values ranged from 0.1-12.7 T- score points. SDC and MIC values could not directly be compared for the same PROMIS measure but SDC values were mostly lower than MIC values.

**Conclusion**

Limited evidence is available on the SDC and MIC of PROMIS measures. More high quality evidence is needed. Data from test-retest reliability studies was not optimally used and should be re-analysed to estimate the SDC. Higher quality anchors and larger sample sizes are needed in MIC studies.

## 167-O. Effectiveness of automated EMR notification for prompting provider intervention for severe depression

### Marco Castro, Mark Vrahas

#### Both authors: Cedars-Sinai Medical Center

##### **Correspondence**: Mark Vrahas (mark.vrahas@cshs.org)

**Objective**

Although there is good evidence that patient-reported outcome measures can be used to improve patient-doctor communication, and can uncover unrecognized problems, they are only useful if physicians use them. The purpose of this study was to determine if simple EMR notifications would prompt providers to address severe depression when present in their patients. In March of 2018 we initiated the routine collection of PROMIS, Physical Function, Pain Interference, and Depression/Mood CATS in our orthopaedic clinics. By May of 2018 we noticed that 2% of patients had Depression CAT scores consistent with severe depression. To address this, we worked with EIS to prepare a Best Practice Alert for patients with severe depression. An automated in-basket message was sent to the nursing triage pool for patients with depression scores greater that 70. The triage nurse forwarded the note to the visit provider notifying the physician of the high score and provided resources for referring the patient to their PCP or a mental health provider. No other notification was provided.

**Methods**

Between October 2018 and March 2020, 282 patients were identified as having severe depression (PROMIS Depression CAT >70), and the responsible physician was made aware as noted above. A retrospective quality review of progress notes to determine if providers documented the high score and provided some intervention.

**Results**

The physicians documented the high score in 63 cases (22%). No mention was made of the depression in 219 cases (78%). Interventions noted for the 63 patients, where the physician documented the high depression score, are noted in table 1.

**Discussion**

A simple Best Practice Alert was not adequate to prompt physicians to document some intervention to address their patient’s depression. Nevertheless, this simple intervention was effective in 22% of cases. In addition, it may be possible that the physician did indeed address the patient’s depression and just did not document it in the chart. The mechanism to provide notification does not disrupt clinic flow in any way, and physician education along with standard macros for documentation may greatly increase the numbers of patients whose depression are being addressed.

**Table 1 (abstract 167-O).** See text for description

Referred to	Count of MRN	%
Mental Health Provider	27	42.86%
Not specified	25	39.68%
Social Worker	5	7.94%
PCP	5	7.94%
Program or Resource	1	1.59%
**Grand Total**	**63**	

## 168-P. Progress in clinical application of patient reported outcomes

### Ying Wang, Zhixia Jiang

#### Both authors: Department of Nursing, Affiliated Hospital of Zunyi Medical University

##### **Correspondence:** Jamie Wang (wyjamie@126.com)

**Objective**

Patient-Reported Outcomes (PROs) refer to the subjective evaluation of the patient's own health status directly from the patient. With the rapid development of medical treatment in recent years, people are becoming more aware of the importance of patient self-reports in clinical evaluation. The research of PROs is more and more clinically carried out. This article is about the progress of clinical research of PROs at home and abroad.

**Methods**

Search domestic and foreign databases to summarize and evaluate relevant literature.

**Results**

A total of 1 958 related documents were retrieved. PROs are widely used in patients with various diseases, such as leukemia patients, lung cancer patients, breast cancer patients, pelvic floor dysfunction patients, femoral head necrosis hip preservation patients, lumbar disc herniation patients, Patients with chronic obstructive pulmonary disease complicated with pulmonary heart disease in Chinese medicine, patients with anatomical total shoulder replacement, patients with hip replacement, and knee replacement. Pay attention to the health-related outcomes reported by patients, listen to the true feelings of patients, provide patients with an understanding of their own health status, adverse reactions that occur during treatment, the impact of physical functions and the impact of different environments on personal and family life. Research hotspots in the field of chronic diseases for several years.

**Conclusions**

With the continuous development and improvement of PROs, its high reliability and simplicity of measurement have been recognized by more and more researchers and clinical practitioners, but the current development and application in China is still in its infancy and requires higher quality research to justified.

## 169-P. Evaluating multiple domains of health in high school athletes with sport-related concussion

### Richelle M. Williams^1^, Rachel S. Johnson,^2^; Alison R. Snyder Valier^3,4,6^; R. Curtis Bay^5^, Tamara C. Valovich McLeod^3,6^

#### ^1^Department of Athletic Training, Drake University, Des Moines, IA; ^2^Department of Kinesiology, University of Georgia, Athens, GA; ^3^Athletic Training Programs, ^4^Department of Research Support; ^5^Department of Interdisciplinary Health Sciences; ^6^ School of Osteopathic Medicine in Arizona, A.T. Still University, Mesa, AZ

##### **Correspondence:** Richelle M. Williams (Richelle.williams@drake.edu)

**Objective**

The objective of this study was to evaluate patient-perceived health-related quality of life (HRQOL) using the Patient-Reported Outcomes Measurement Information System (PROMIS) Pediatric-25 subscale in adolescent patients throughout concussion recovery and to describe the impact of sport-related concussion history on HRQOL.

**Methods**

This study included a convenience sample of male and female interscholastic students from nine high school athletic training facilities who were participating in sports. Patients who sustained isolated medically diagnosed concussions from sports participation were administered the PROMIS Pediatric-25 scale at days 3 and 10 post-concussion and at return-to-play (RTP). The following subscales were used for analysis: Physical Function Mobility (PFM), Anxiety (ANX), Depression (DS), Fatigue (FTG), Pain Interference (PI), and Peer Relationships (PR). Self-reported concussion history (yes/no) was collected. Generalized estimating equations were used for primary PROMIS analysis (p<.05) and summary statistics were reported as means and 95% CI. Ceiling and floor effects (more than 15% of patients reporting the highest or lowest score on the subscale, respectively) are reported.

**Results**

Seventy patients completed the study (51 males, 7 females, 12 unreported, age=15.7±0.9 years, height=174.6±8.4cm, mass=72.8±14.8 kg, grade=10.0±0.9 level). For the Pediatric-25 subscales, the severity of problems associated with PFM, ANX, DS, FTG, and PI were highest 3 days post-concussion, decreasing at 10 days post and RTP (all *P*<.05). No differences were found between days 3 and 10 for PR scores, but improvements were identified at RTP (*P*<.05). Pediatric-25 subscale scores at the three measurements were not statistically associated with concussion history (all *P*>.05). Ceiling and floor effects were present in all subscales throughout each time point, except for PFM (14.7%) and PI (11.8%) at day 3 post-injury.

**Conclusions**

Patients who suffered a concussion improved from day 3 through RTP on multiple health domains including function, anxiety, depression, fatigue, and pain. Importantly, HRQOL improved with time since injury; with between 41-79% of patients reaching the instruments’ best score by day 10 post-injury. Serial assessment of health domains is needed to ensure recovery occurs, interventions are provided when deficits exist, and clinicians can use that information to inform clinical decisions.

## 170-P. Psychometric proprieties of PROMIS Anxiety and Depression Short Forms among breast cancer patients in China

### Changrong Yuan, Qingmei Huang, Tingting Cai, Fulei Wu

#### All authors: School of Nursing, Fudan University, Shanghai 200032, China

##### **Correspondence:** Changrong Yuan (yuancr@fudan.edu.cn)

**Objective**

The patient-reported outcomes measurement information system (PROMIS) are developed to assess patient-reported health outcomes for adults and children living with chronic conditions. The adult PROMIS anxiety and depression short forms (SF) have been translated into Chinese and applied in women with breast cancer in China. This study aimed to evaluate the PROMIS anxiety and depression short forms 8a among patients with breast cancer in China.

**Methods**

Internal reliability was evaluated using Cronbach’s alpha coefficient. Besides, scale dimensionality was examined using confirmatory factor analysis (CFA). In addition, concurrent validity was evaluated using correlations between PROMIS anxiety and depression short forms and breast cancer quality of life scores (assessed by Functional Assessment of Chronic Illness Treatment- Breast). Both classical (CTT) and modern (IRT) psychometric methods were used to evaluate the items following the PROMIS validation scientific standards. Known-groups validity was evaluated by comparing T-score differences among patient groups regarding early cancer stage and later cancer stage. In addition, the differential item functioning (DIF), item parameters and scale information curve were analyzed.

**Results**

A final sample comprised 975 breast cancer patients. The Chinese version of PROMIS anxiety-SF and depression-SF both demonstrated good internal consistency reliability. Unidimensionality of the two measures were supported by CFA. The anxiety and depression T-scores across patient group with later cancer stage were significantly higher than those in the early group, which indicated good known-groups validity. According to the IRT item parameters, the item discrimination parameters for anxiety-SF ranged from 1.00-6.32, and the item threshold parameters ranged from - 1.39-2.38. As for depression-SF, the item discrimination parameters and item threshold parameters ranged from 1.00-8.63, and -1.39-2.60, respectively.

**Conclusions**

The Chinese version of adult PROMIS anxiety-SF and depression-SF showed satisfactory psychometric proprieties among breast cancer patients. Additional research in a more diverse sample is necessary to verify the results of this study.

## 171-P. Psychometric evaluation of PROMIS-Social Relationships Short Forms 4a among patients with breast cancer in China

### Tingting Cai, Changrong Yuan, Qingmei Huang, Fulei Wu

#### All authors: School of Nursing, Fudan University, 305 Fenglin Road, Shanghai 200032, China

##### **Correspondence**: Changrong Yuan (yuancr@fudan.edu.cn)

**Objective**

In recent years, patient-reported outcomes measurement information system (PROMIS) has expanded widely as recognition of their usefulness and effectiveness for routine measurement of health and well-being in adults and children. The adult PROMIS Social Relationships short forms 4a have been translated into Chinese based on standard translation methodology. The aim of this study was to validate the Chinese version of the adult PROMIS Social Relationships short 4a among breast cancer patients.

**Methods**

A cross–sectional research design was adopted using the Emotional Support, Informational Support, and Instrumental Support subdomains, which were short forms of adult PROMIS Social Relationships. Web-based and paper questionnaire administration was conducted with a variety of Chinese respondent samples during 2018–2020. Analyses included Cronbach’s alpha, confirmatory factor analysis (CFA), classical test theory (CTT) modeling, item response theory (IRT) modeling and other psychometric methodologies.

**Results**

A total of 965 women with breast cancer in China was recruited and completed the investigations. The Chinese version of adult PROMIS Social Relationships short forms 4a showed satisfactory psychometric proprieties among the patients. Emotional Support, Informational Support, and Instrumental Support showed good internal consistency reliability and validity in this study. Unidimensionality and good known-groups validity of the short forms were supported by the results. In addition, the IRT item parameters further demonstrated the item discrimination parameters for Emotional Support ranged from 1.00-5.27, and the item threshold parameters ranged from -1.76-1.39. With regard to Informational Support, the item discrimination parameters and item threshold parameters ranged from 1.00-6.66, and -1.91-1.39, respectively. As for Instrumental Support, the data ranged from 1.00-4.47, and -2.03-1.39 accordingly.

**Conclusions**

Adult PROMIS Social Relationships short forms 4a of Chinese version are demonstrated to be feasible, valid, and reliable in breast cancer patients in China. Further research in a more diverse sample and with different study design are encouraged to verify the results in the current study.

## 172-P. Using cognitive interviewing to adapt PROMIS^®^ Measure Items of Self-efficacy for managing chronic conditions in Chinese cross-cultural translation

### Dan Zhao^1^, Linning Yang^2^, Yan Jin^3^, Jiehui Xu^3^, Ting Zhang^3^, Xiuqun Yuan^3^, Danfeng Zha^3^, Yan Yang^1^

#### ^1^School of Nursing, Medical College, Soochow University, Suzhou, China; Renji Hospital affiliated to Shanghai Jiaotong University School of Medicine, Shanghai, China; ^2^School of Nursing, Shanghai Jiaotong University, Shanghai, China; ^3^Renji Hospital affiliated to Shanghai Jiaotong University School of Medicine, Shanghai, China

##### **Correspondence:** Dan Zhao, (zd_2527@163.com)

**Objective**

To use cognitive interviewing techniques to evaluate the comprehension, wording and format of PROMIS^®^ Self- efficacy items among Chinese living with chronic conditions after FACIT forward-backward translation and then to revise items based on subjects’ feedback.

**Methods**

We conducted 25 cognitive interviews of native Chinese speakers with diversity range of chronic conditions (e.g., diabetes, hypertension, obesity, inflammatory bowel disease, hepatic sclerosis, peptic ulcer, and cardiovascular disease) on 137 items of self-efficacy. Retrospective probing was chosen due to the self-administered questionnaire. Audio recordings were used to cover the gaps in the handwritten comments and were not transcribed verbatim. Questionnaire Appraisal System (QAS-99) was used to code the text data. A series of feedback from subjects were compiled. Then the experts and translation group reviewed the data and decided whether to revise or rewrite each potential item.

**Results**

The appraisal from the cognitive interviews identified most items were easy understanding. Meanwhile, some issues were encountered in the lengthy, awkward, syntax of the wording. 5 out of 25 emotions items, 8 out of 35 daily activities items, 6 out of 26 medicine and treatment items, 2 out of 23 social interaction items and 4 out of 28 symptoms items were found to have poor comprehension mainly for the reason of wording. And 7 of 137 items had to rewritten because of culturally inappropriate. All these items were revised base on the subjects’ feedback, linguistic rules and the expert suggestions.

**Conclusions**

Cognitive interviewing is a useful method in conducting quality assessment of already developed questionnaire and adapting items in the context of simplified Chinese. Results showed that the revised version of PROMIS self-efficacy items is conceptually, culturally and semantically equivalent to the original. Future work related to Chinese version psychometric properties validation of PROMIS self-efficacy for managing chronic conditions can now be initiated.

## 173-P. Development of a Pediatric PROMIS mini-program based on Wechat application for children and adolescents

### Wen Zhang, Qingmei Huang, Yueshi Huang, Changrong Yuan

#### All authors: School of Nursing, Fudan University, Shanghai, China

##### **Correspondence**: Changrong Yuan (yuancr@fudan.edu.cn)

**Objective**

This study was to develop a smartphone application Wechat-based mini-program to enable children and adolescents aged 5-17 years old and their proxies to assess pediatric patients’ quality of life-related symptoms and functions using Pediatric PROMIS profile-25.

**Methods**

A multidisciplinary team including researchers, clinical professionals and software engineers was formed to discuss the contents, structures, and functions of the program and its administrative portal, to make longitudinal assessment and data management more efficient, and the interface more user-friendly. Several rounds of joint sessions and modifications were performed among the team to assure the quality of the program during the development.

**Result**

The Wechat mini-program ‘PROMIS Assessment’ for pediatric patients aged 8-17 years old and proxies of all 5-17 year-old children was created. Pediatric PROMIS profile-25 involving seven dimensions (depressive symptoms, anxiety, fatigue, physical activity-mobility, peer relationship, pain interference, and pain intensity) was used for QoL assessment. Demographic information of both children and proxies and patients’ clinical information were also included. Outcomes were shown with graphs to users in the ‘Disease Management’ and ‘Symptoms Management’ parts. Voice assistant, cartoon interface, and virtual animal raise system were used to help increase children’s understanding and compliance.

**Conclusion**

The smartphone mini-program based on Wechat and its administration portal were developed to assess and collect quality of life outcomes of pediatric patients using brief items. It helps parents self-monitor their children’s disease progress and tendency of symptoms and functions, as well as giving medical professionals patient-reported outcomes to help make more targeted medical decisions.

## 174-P. The Patient-Reported Outcomes Measurement Information System (PROMIS®) Physical Function measures in adults – A systematic review of measurement properties

### Valentijn J. Zonjee^1^, Inger L. Abma^2^, M.J. de Mooij^1^, Sander M. van Schaik^1^, Renske M. Van den Berg-Vos^1^, Caroline B. Terwee^3^, Leo D. Roorda^4^

#### ^1^OLVG Amsterdam, Department of Neurology, Amsterdam, The Netherlands; ^2^IQ healthcare, Radboudumc, Nijmegen, The Netherlands; ^3^Amsterdam UMC, Vrije Universiteit Amsterdam, Department of Epidemiology and Biostatistics, Amsterdam Public Health Research Institute; ^4^Amsterdam Rehabilitation Research Center | Reade, Amsterdam, The Netherlands

##### **Correspondence:** Valentijn Zonjee (v.j.zonjee@olvg.nl)

**Objective**

This study aims to systematically review and critically appraise the measurement properties of Patient-Reported Outcomes Measurement Information System (PROMIS®) Physical Function (PF) measures in adults.

**Methods**

MEDLINE and EMBASE were searched for studies on PROMIS-PF measurement properties. Studies were included if the aim of the study was to evaluate at least one measurement property of a PROMIS-PF measure in adults. No restrictions were made with respect to language, study design and the type of application of the measure. Following the COnsensus-based Standards for the selection of health Measurement INstruments (COSMIN) methodology, Risk of bias was assessed using the COSMIN Risk of Bias checklist. The results per measurement property were quantitatively or qualitatively pooled and the quality of evidence was determined.

**Results**

The database searches identified 1086 unique studies. After title and abstract screening by two independent reviewers, 284 studies were deemed eligible for full-text screening.

**Conclusions**

This study will systematically summarize, pool and appraise the measurement properties of all PROMIS-PF measures that are currently available.

